# Supplementary figures and images for: Phenylhydrazone-based endoplasmic reticulum proteostasis regulator compounds with enhanced biological activity
Source: eLife. 2026 Jan 26;14:RP107000. doi: 10.7554/eLife.107000 (PMC12834500; doi:10.7554/eLife.107000)

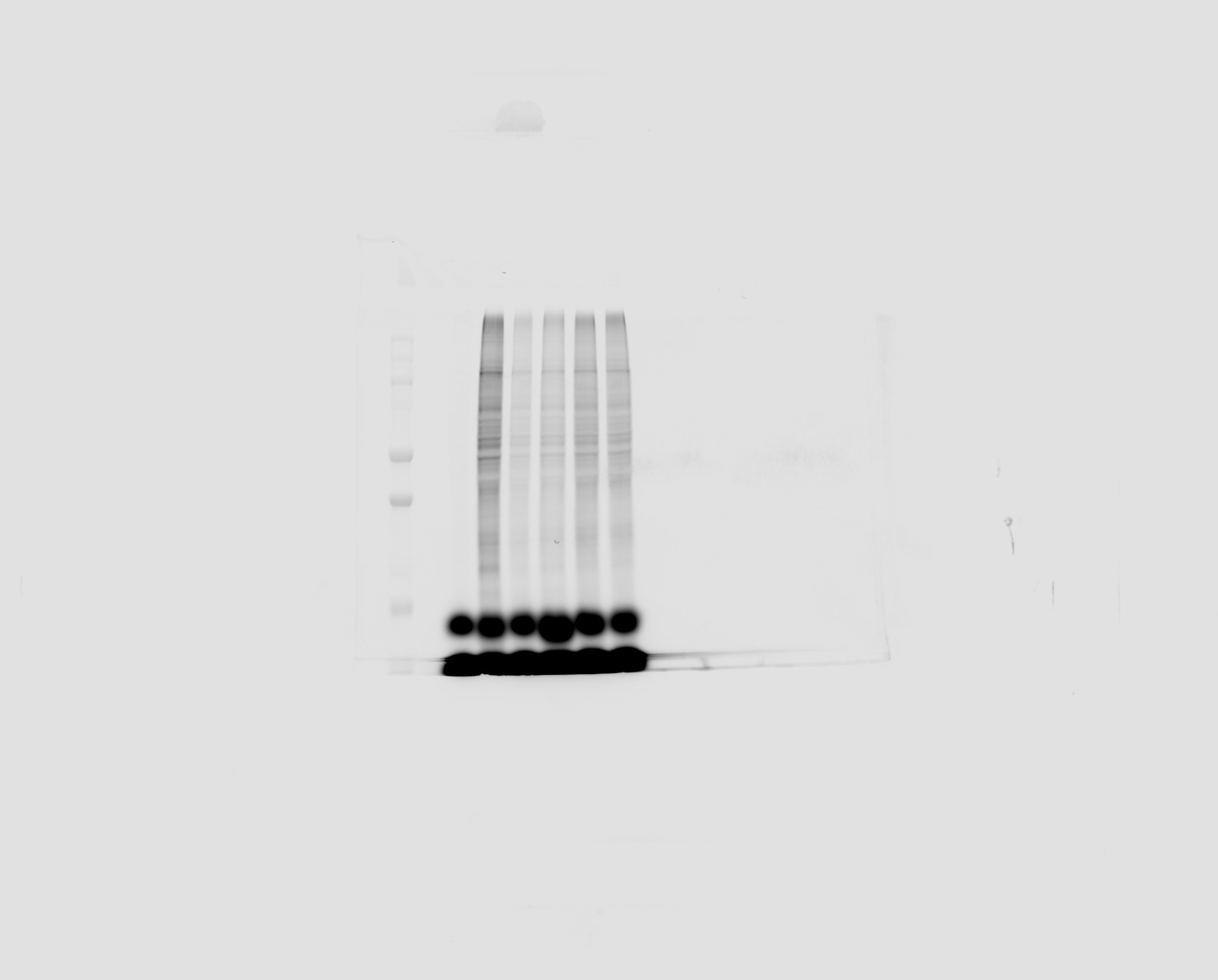

Supplement: Figure 1—source data 2. [file elife-107000-fig1-data2.zip › Figure 1 - source data 2/Figure 1G raw.tif]

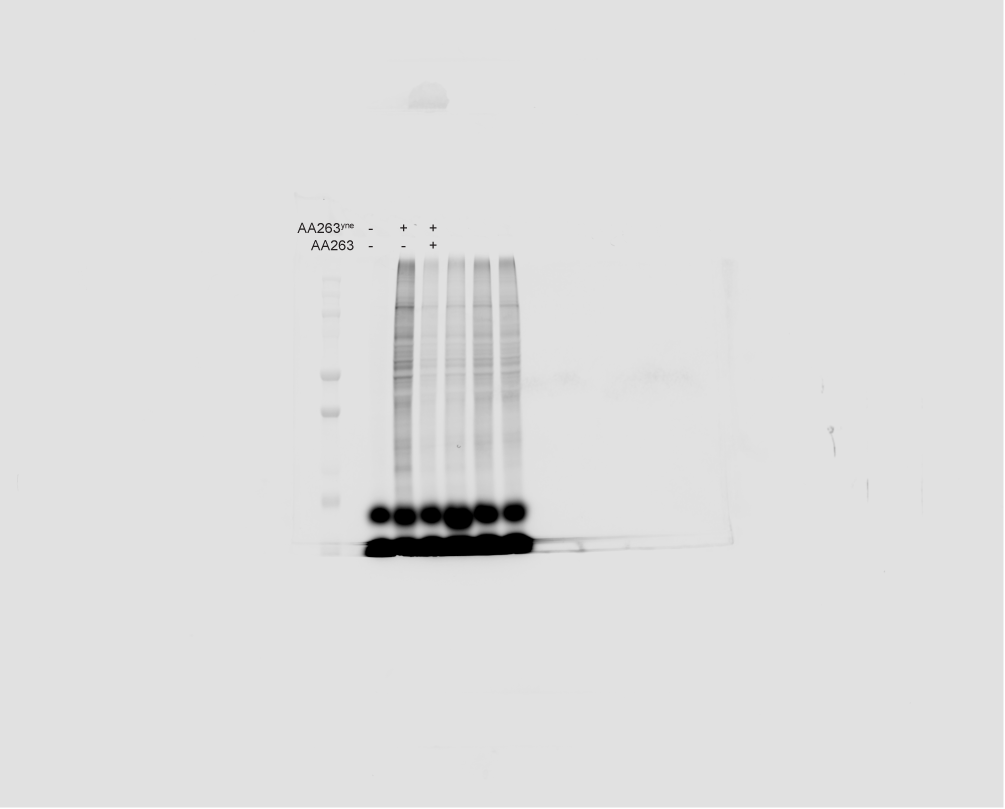

Supplement: Figure 1—source data 3. [file elife-107000-fig1-data3.zip › Figure 1 - source data 3/Figure 1G.tif]

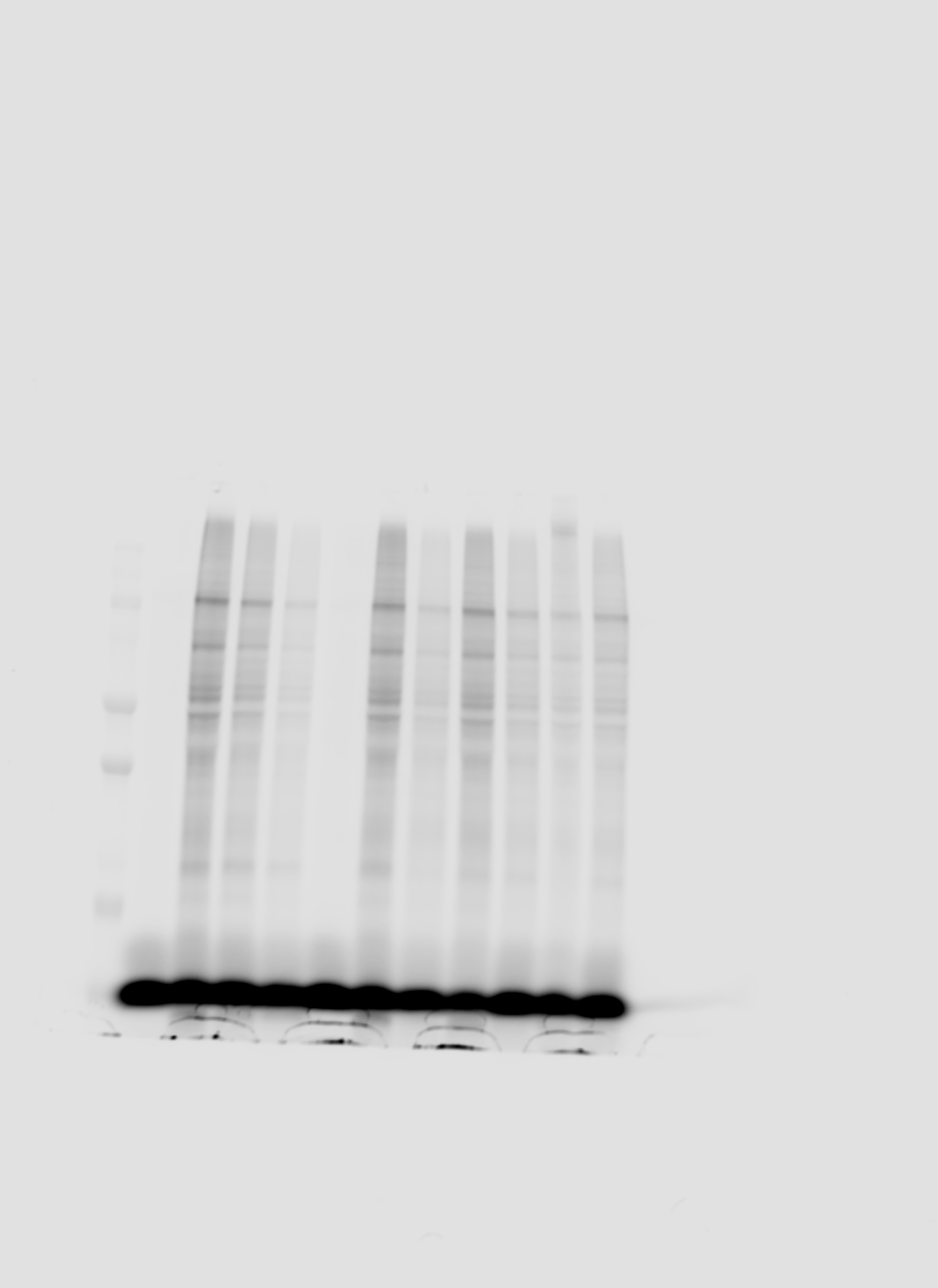

Supplement: Figure 1—figure supplement 1—source data 2. [file elife-107000-fig1-figsupp1-data2.zip › Figure 1 - figure supplement 1 - source data 2/Figure S1C raw.tif]

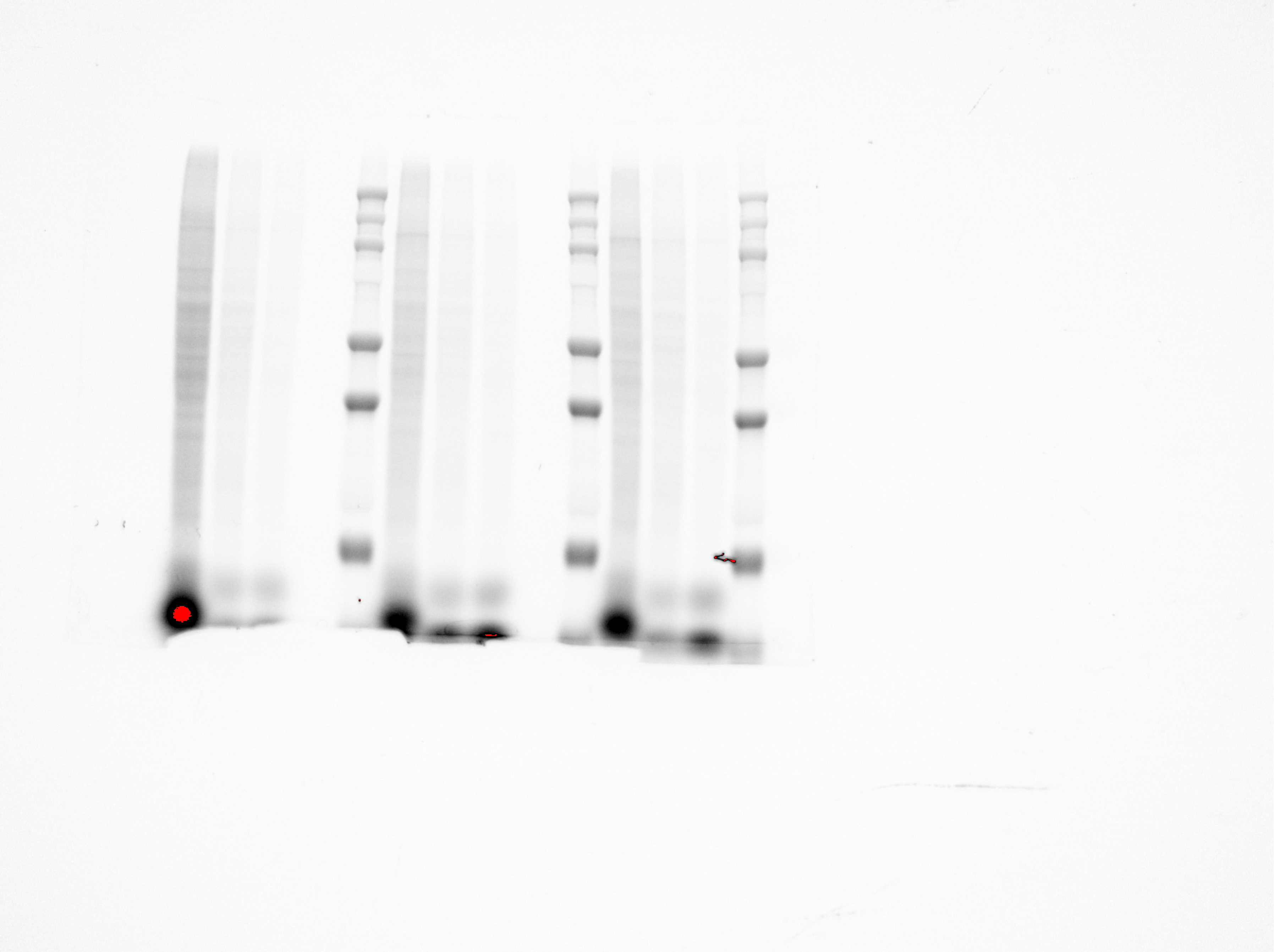

Supplement: Figure 1—figure supplement 1—source data 2. [file elife-107000-fig1-figsupp1-data2.zip › Figure 1 - figure supplement 1 - source data 2/Figure S1D raw.tif]

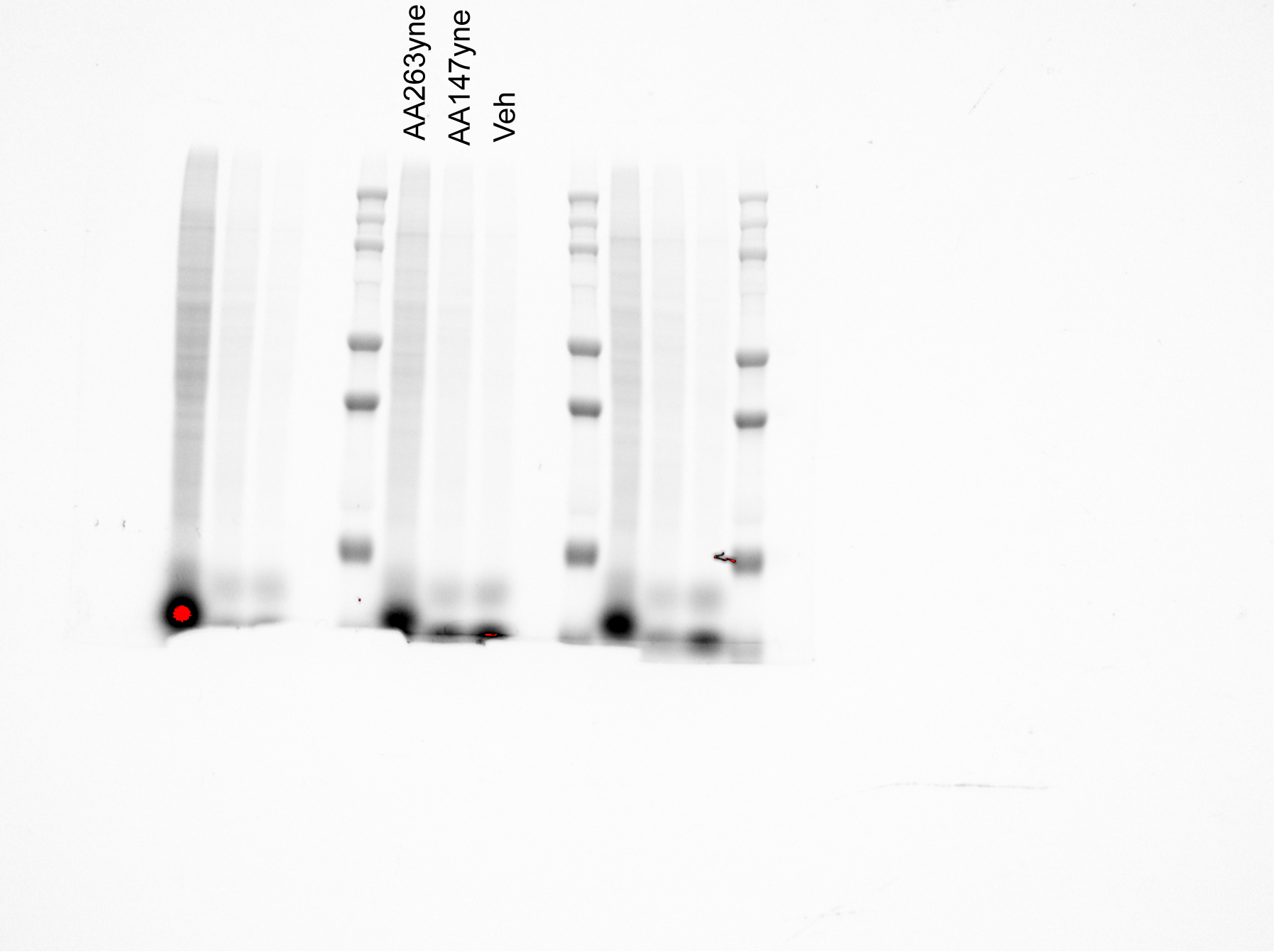

Supplement: Figure 1—figure supplement 1—source data 3. [file elife-107000-fig1-figsupp1-data3.zip › Figure 1 - figure supplement 1 - source data 3/Figure S1D.tif]

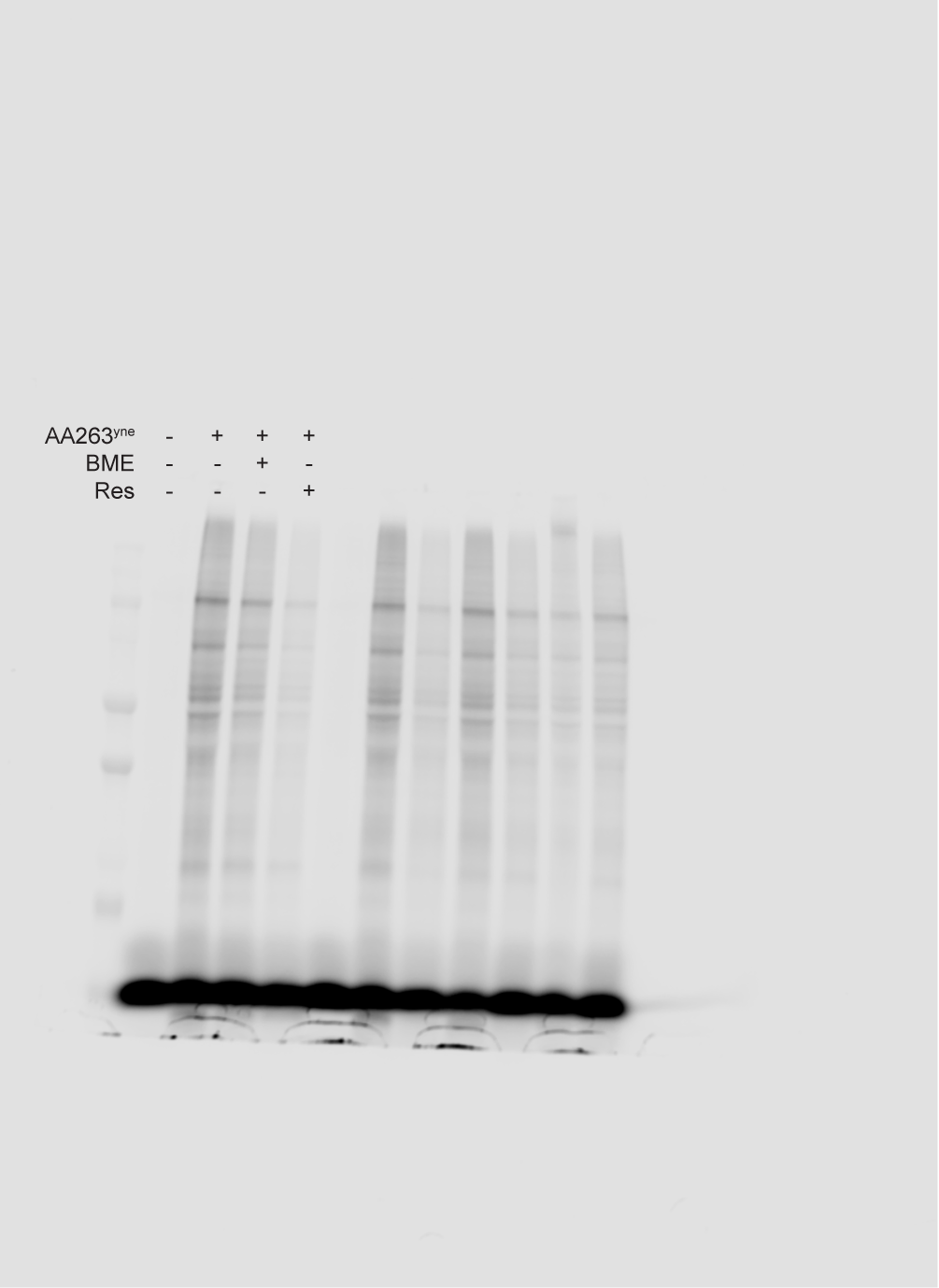

Supplement: Figure 1—figure supplement 1—source data 3. [file elife-107000-fig1-figsupp1-data3.zip › Figure 1 - figure supplement 1 - source data 3/Figure S1C.tif]

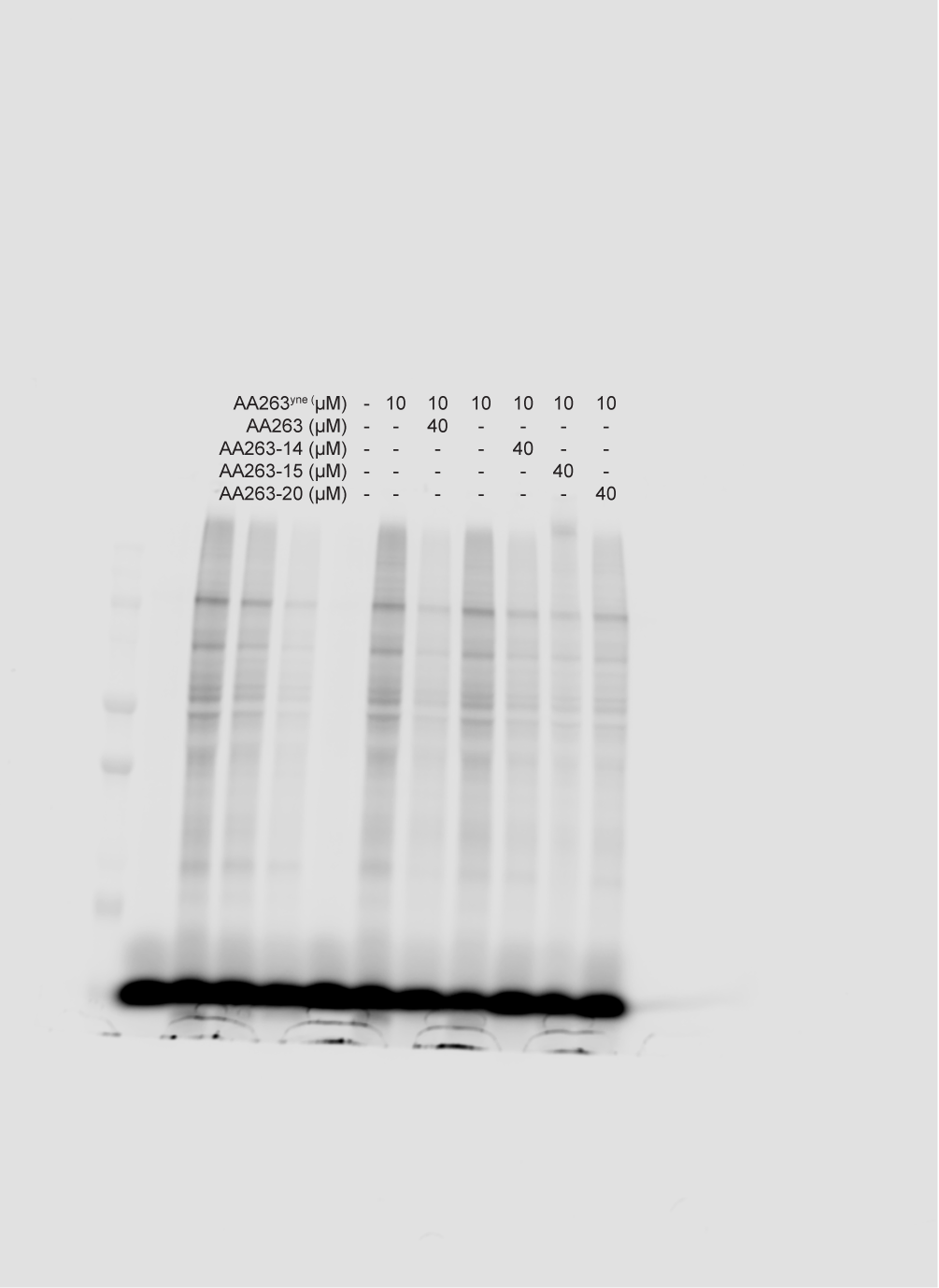

Supplement: Figure 3—figure supplement 1—source data 3. [file elife-107000-fig3-figsupp1-data3.zip › Figure S3B.tif]

Fig 5A. Surface  $\gamma 2$

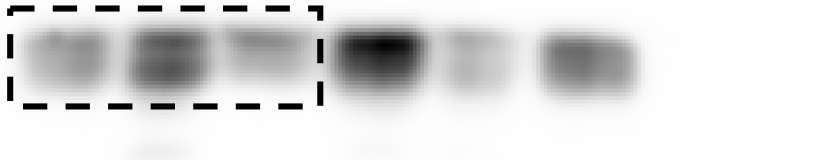

Fig 5A.  $\text{Na}^+/\text{K}^+$  ATPase

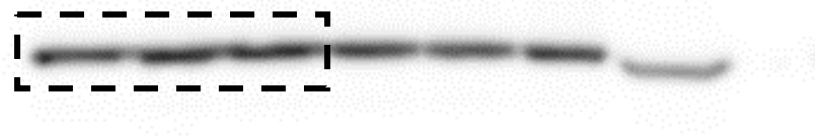

Fig 5B. Surface  $\gamma 2$

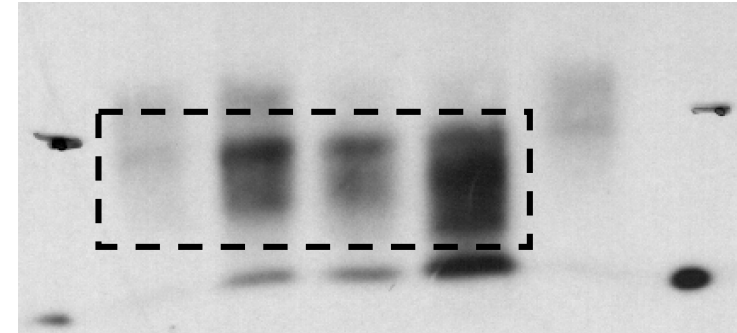

Fig 5B.  $\text{Na}^+/\text{K}^+$  ATPase

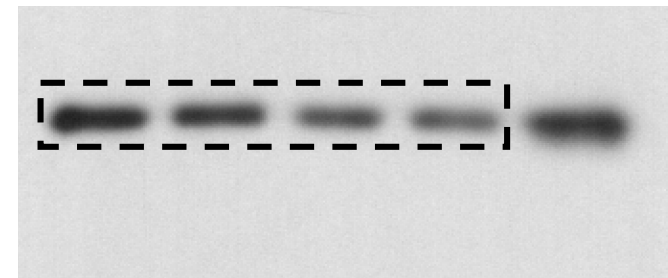

Supplement: Figure 5—source data 2. [file elife-107000-fig5-data2.zip › Figure 5 - source data 2/Figure 5 Source data 1.pdf]

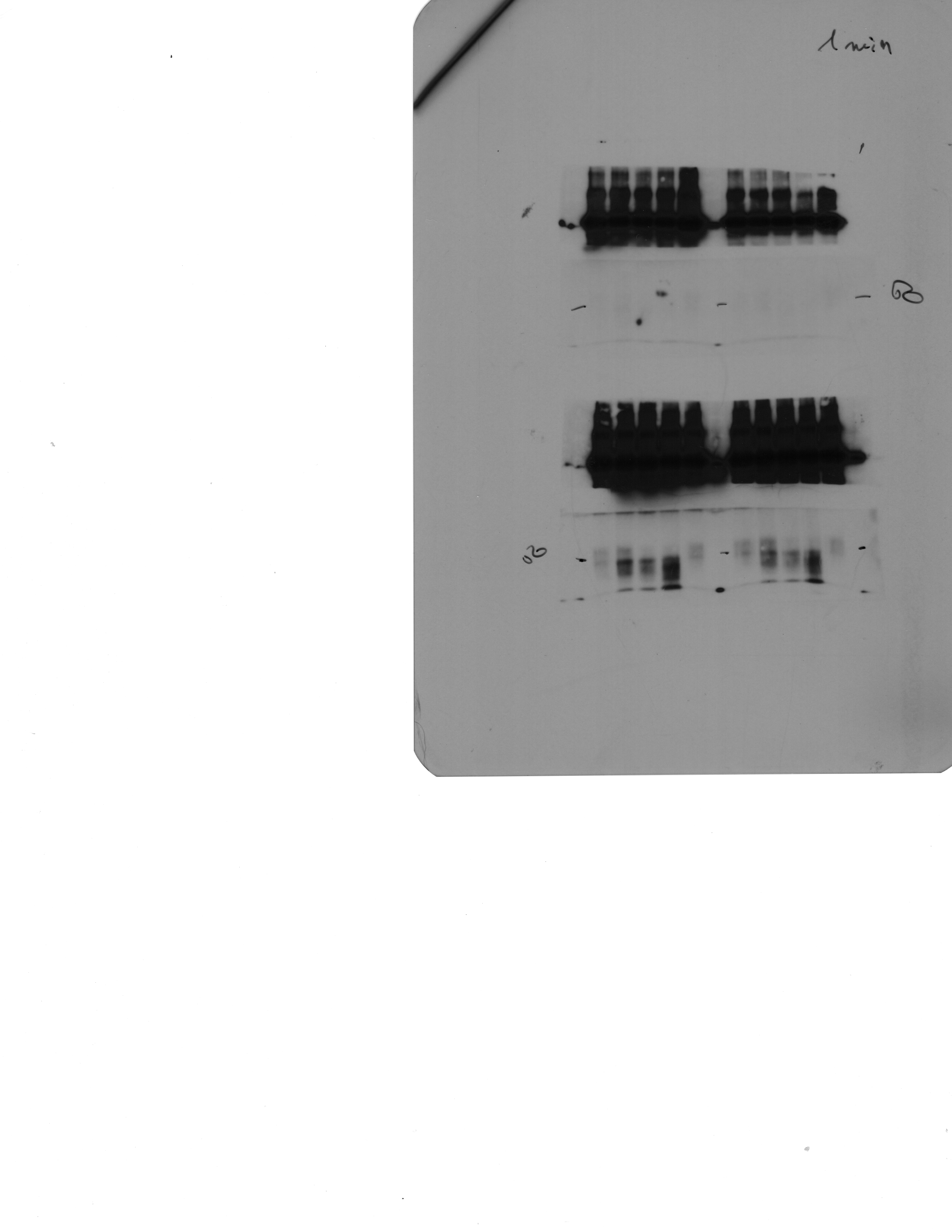

Supplement: Figure 5—source data 3. [file elife-107000-fig5-data3.zip › Figure 5 - source data 3/Fig 5B Surface gamma2.tif]

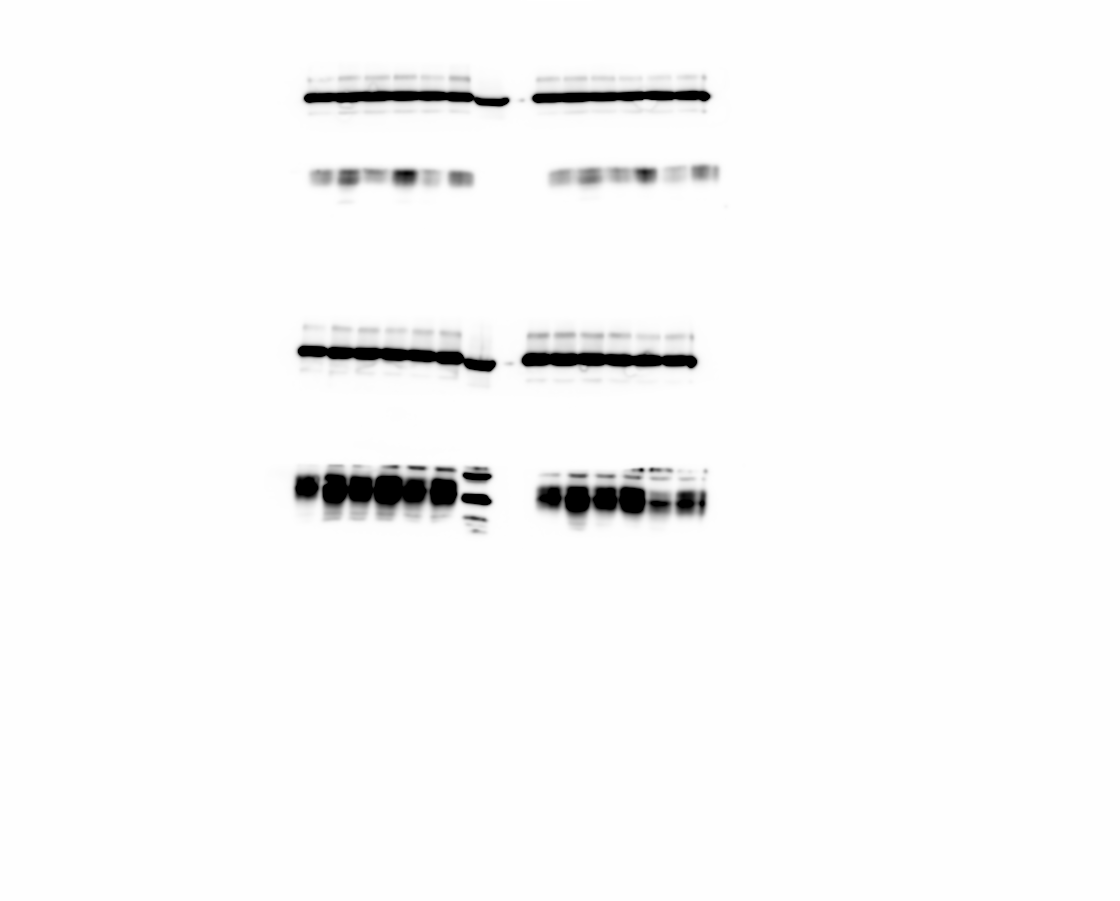

Supplement: Figure 5—source data 3. [file elife-107000-fig5-data3.zip › Figure 5 - source data 3/Fig 5A surface gamma2.tif]

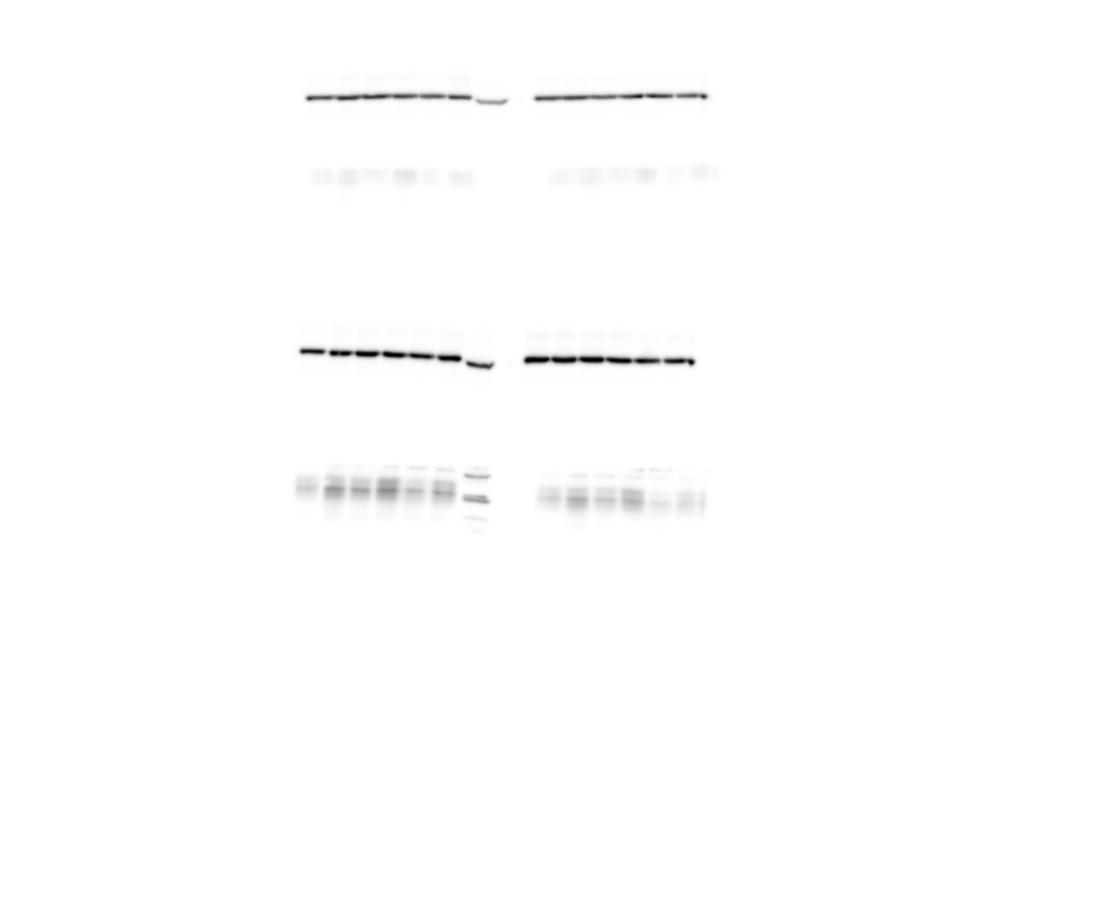

Supplement: Figure 5—source data 3. [file elife-107000-fig5-data3.zip › Figure 5 - source data 3/Fig 5A ATPase.tif]

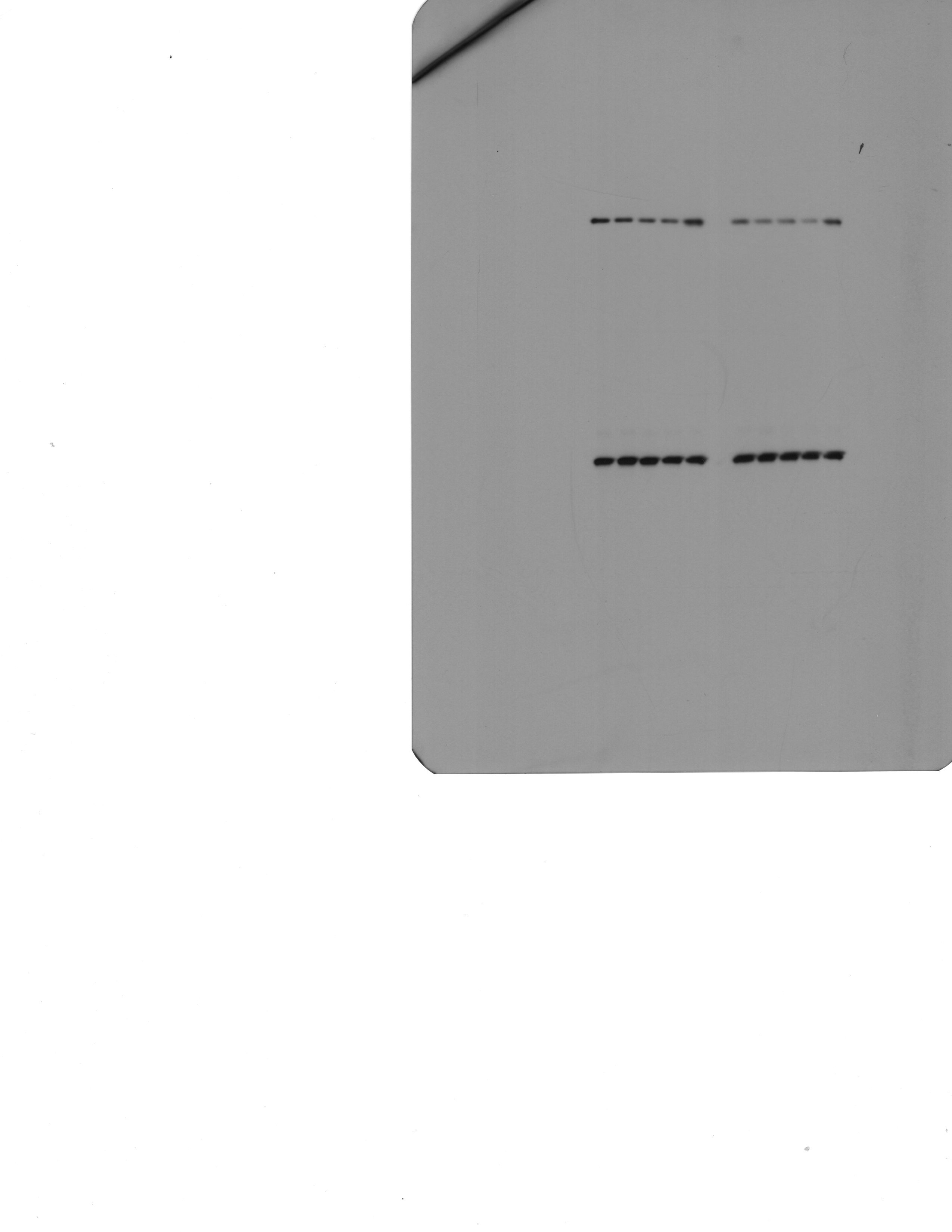

Supplement: Figure 5—source data 3. [file elife-107000-fig5-data3.zip › Figure 5 - source data 3/Fig 5B ATPase.tif]

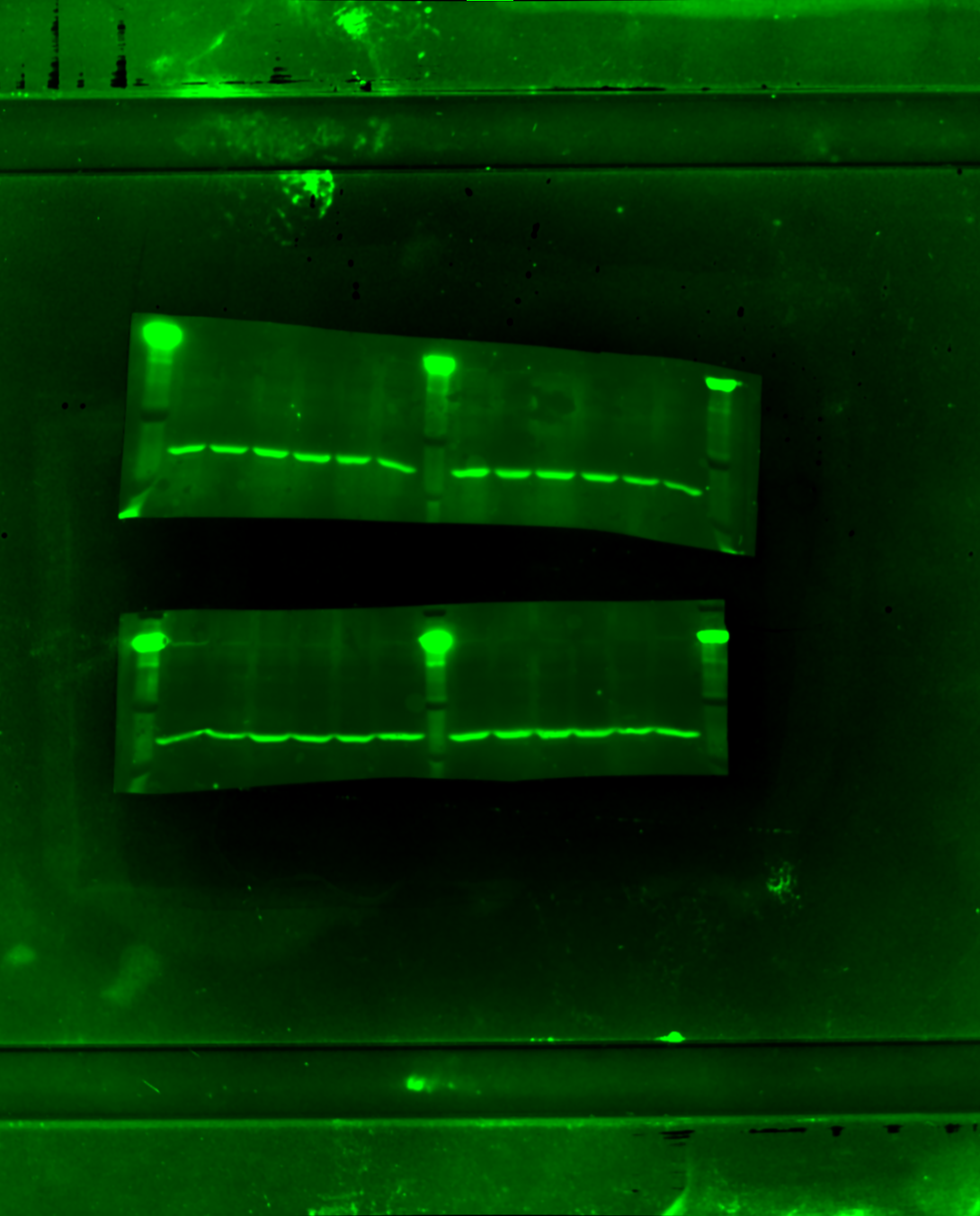

Supplement: Figure 5—figure supplement 1—source data 3. [file elife-107000-fig5-figsupp1-data3.zip › Figure 5 - figure supplement 1-source data 3/Fig S5C RG AA-263yne actin.tif]

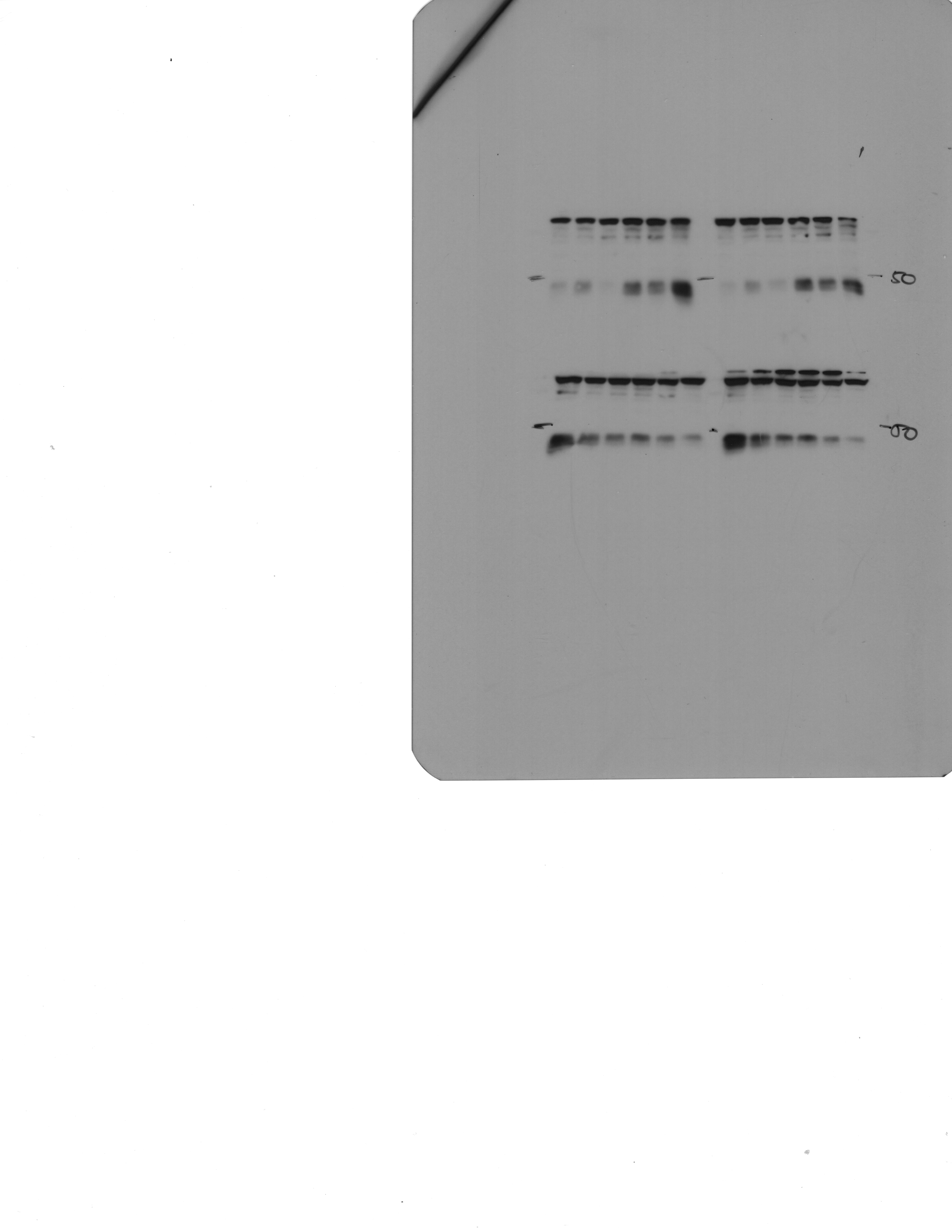

Supplement: Figure 5—figure supplement 1—source data 3. [file elife-107000-fig5-figsupp1-data3.zip › Figure 5 - figure supplement 1-source data 3/Fig S5C RG AA-263yne total gamma2.tif]

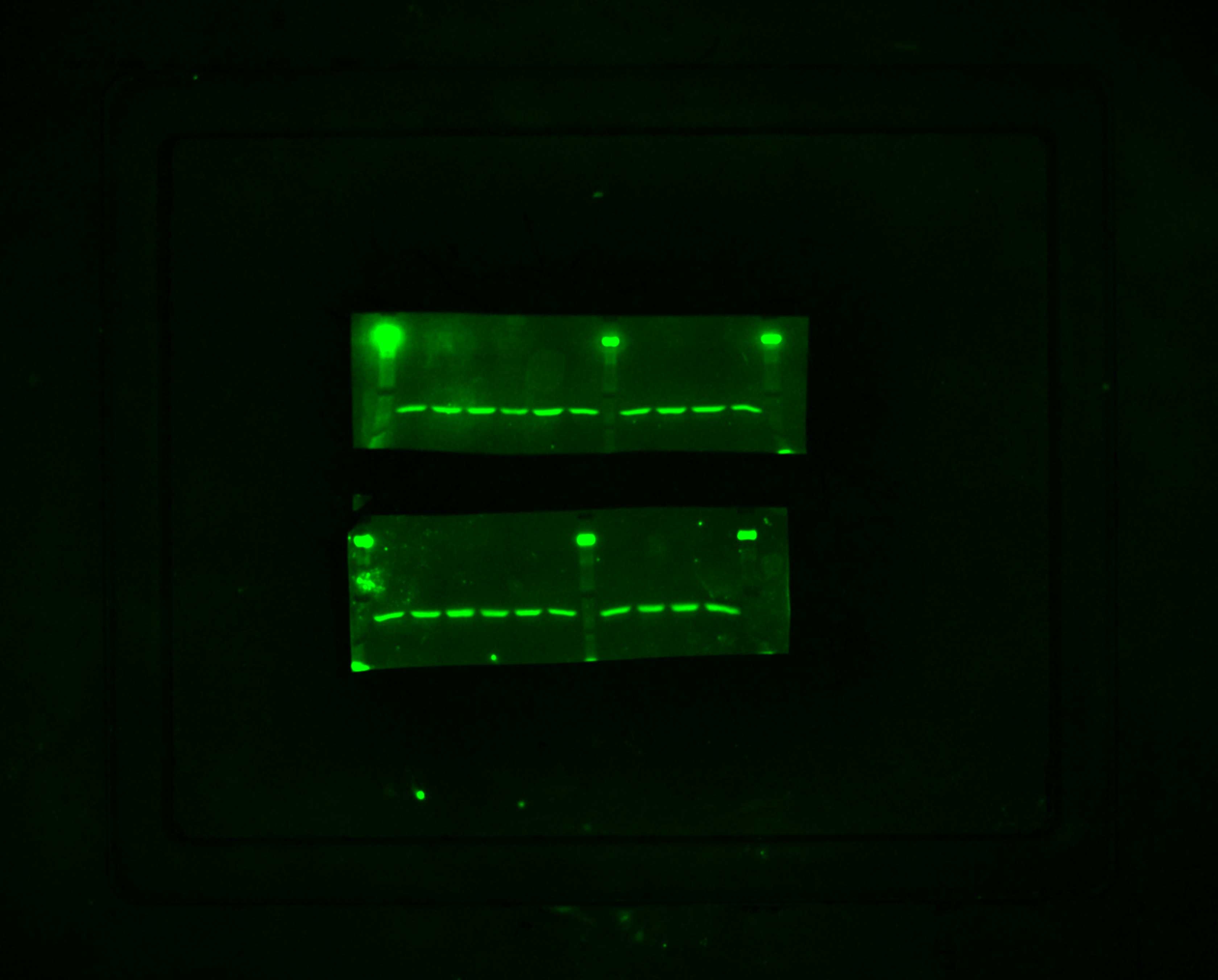

Supplement: Figure 5—figure supplement 1—source data 3. [file elife-107000-fig5-figsupp1-data3.zip › Figure 5 - figure supplement 1-source data 3/Fig S5A actin.tif]

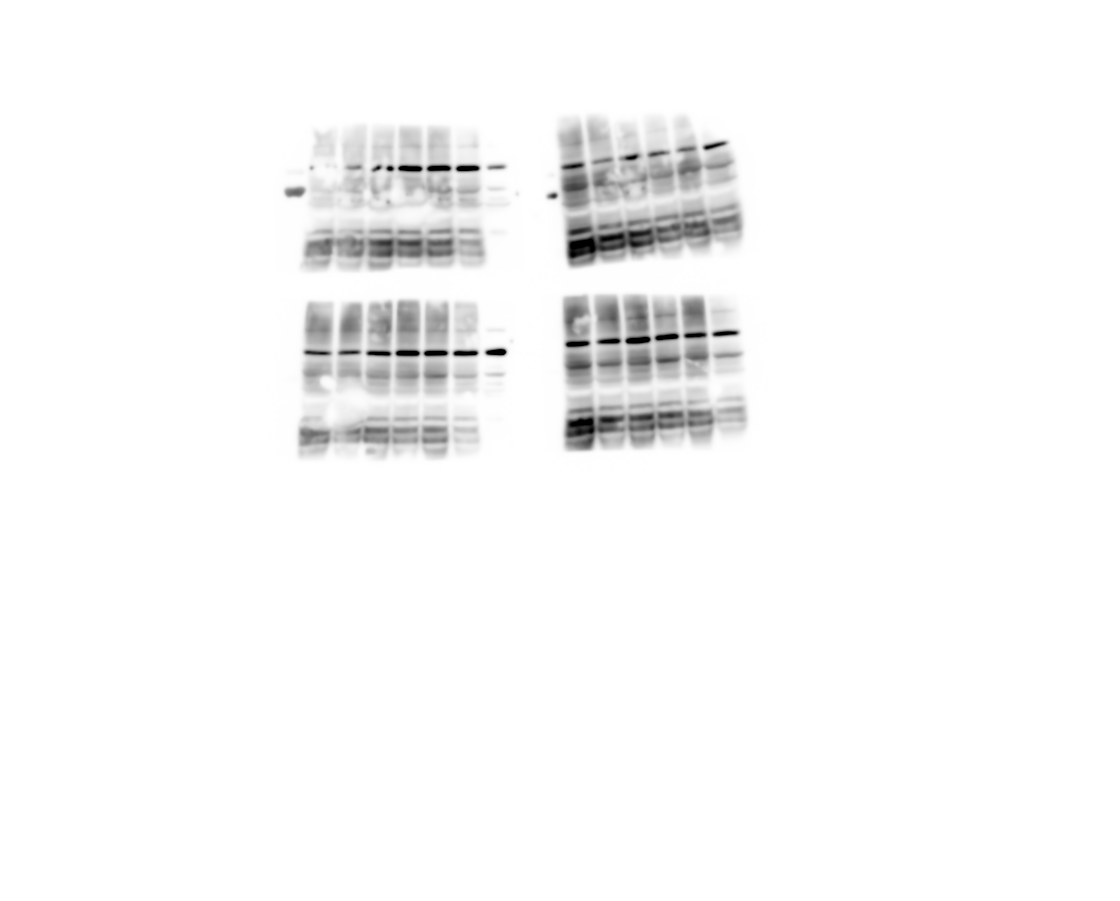

Supplement: Figure 5—figure supplement 1—source data 3. [file elife-107000-fig5-figsupp1-data3.zip › Figure 5 - figure supplement 1-source data 3/Fig S5C WT total gamma2.tif]

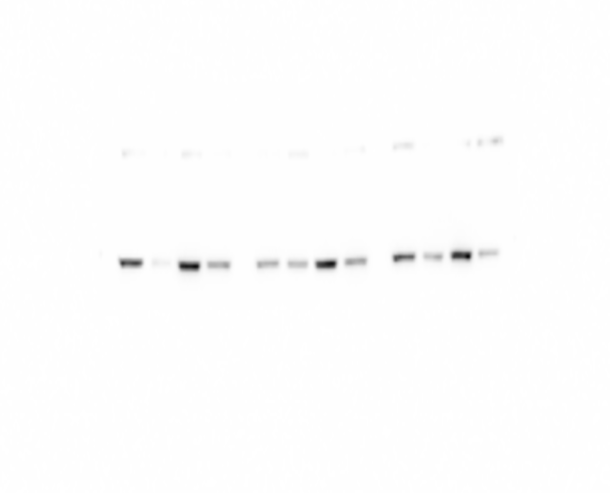

Supplement: Figure 5—figure supplement 1—source data 3. [file elife-107000-fig5-figsupp1-data3.zip › Figure 5 - figure supplement 1-source data 3/Fig S5B BiP.tif]

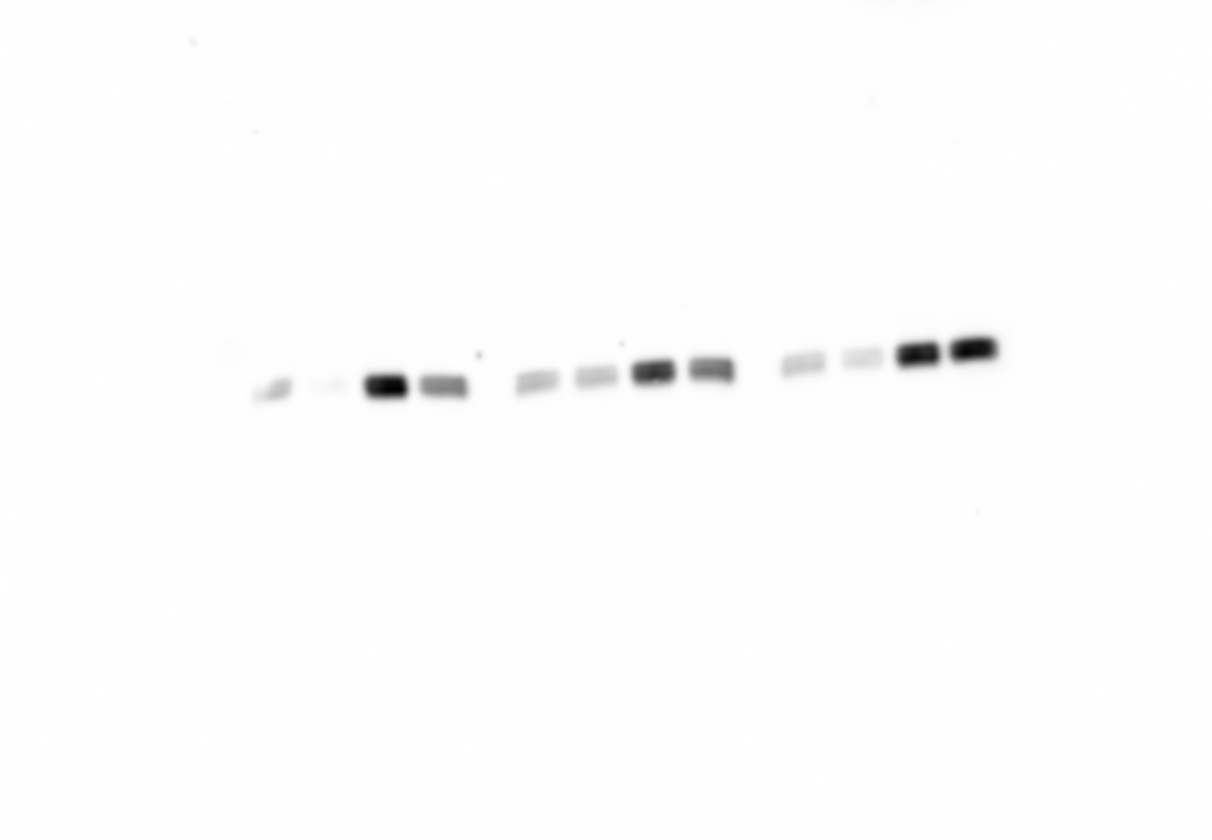

Supplement: Figure 5—figure supplement 1—source data 3. [file elife-107000-fig5-figsupp1-data3.zip › Figure 5 - figure supplement 1-source data 3/Fig S5B gamma2.tif]

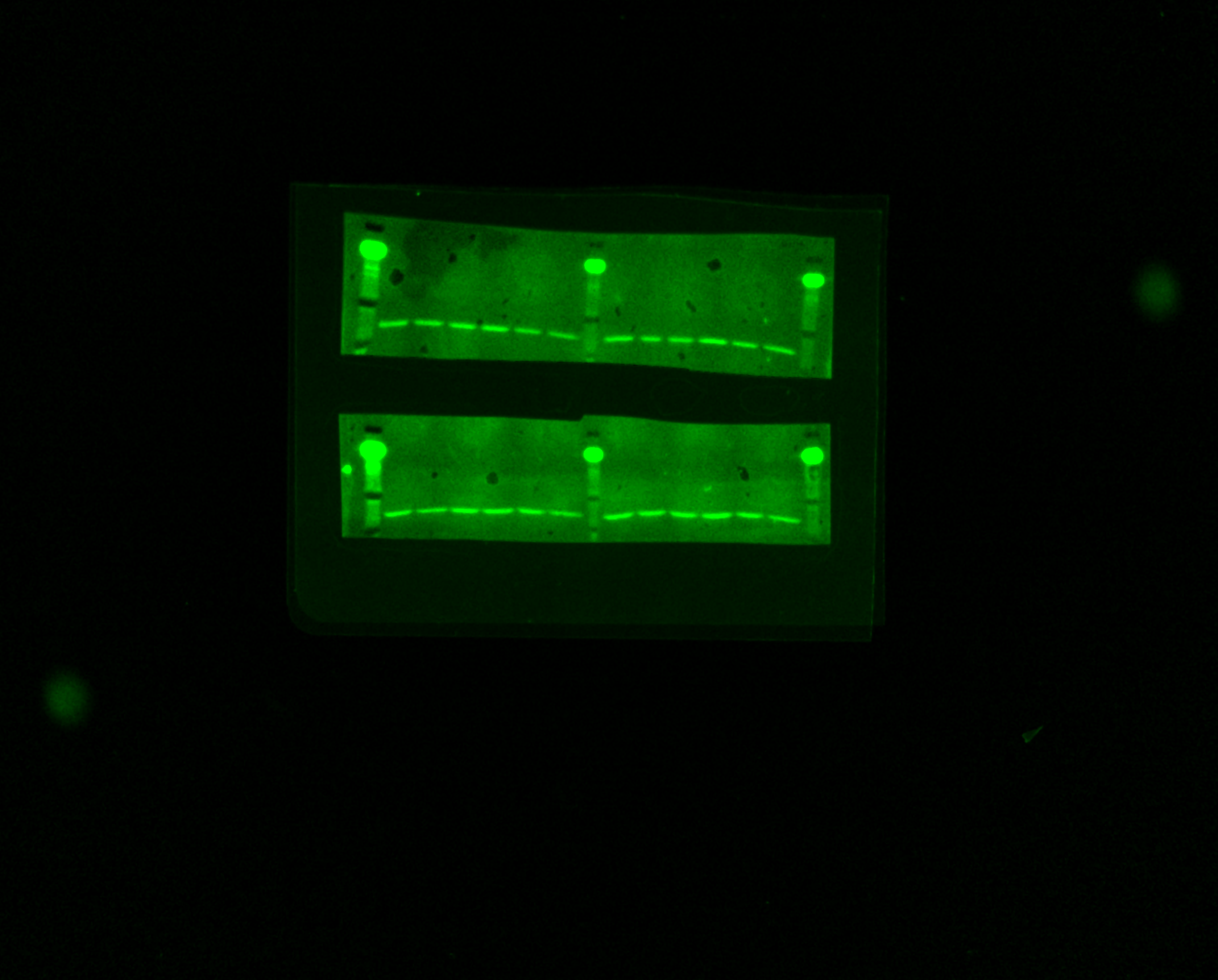

Supplement: Figure 5—figure supplement 1—source data 3. [file elife-107000-fig5-figsupp1-data3.zip › Figure 5 - figure supplement 1-source data 3/Fig S5C RG actin.tif]

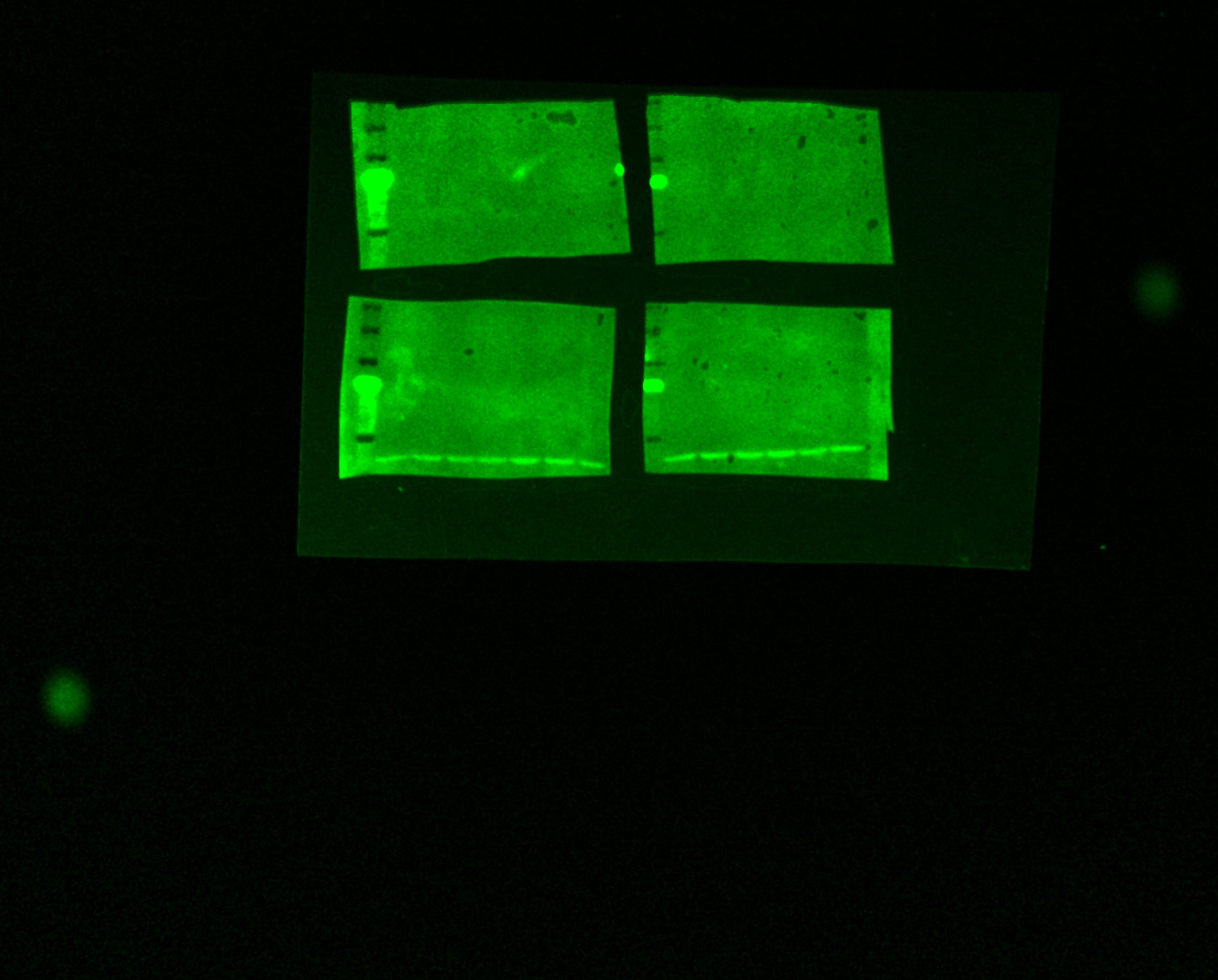

Supplement: Figure 5—figure supplement 1—source data 3. [file elife-107000-fig5-figsupp1-data3.zip › Figure 5 - figure supplement 1-source data 3/Fig S5C WT actin.tif]

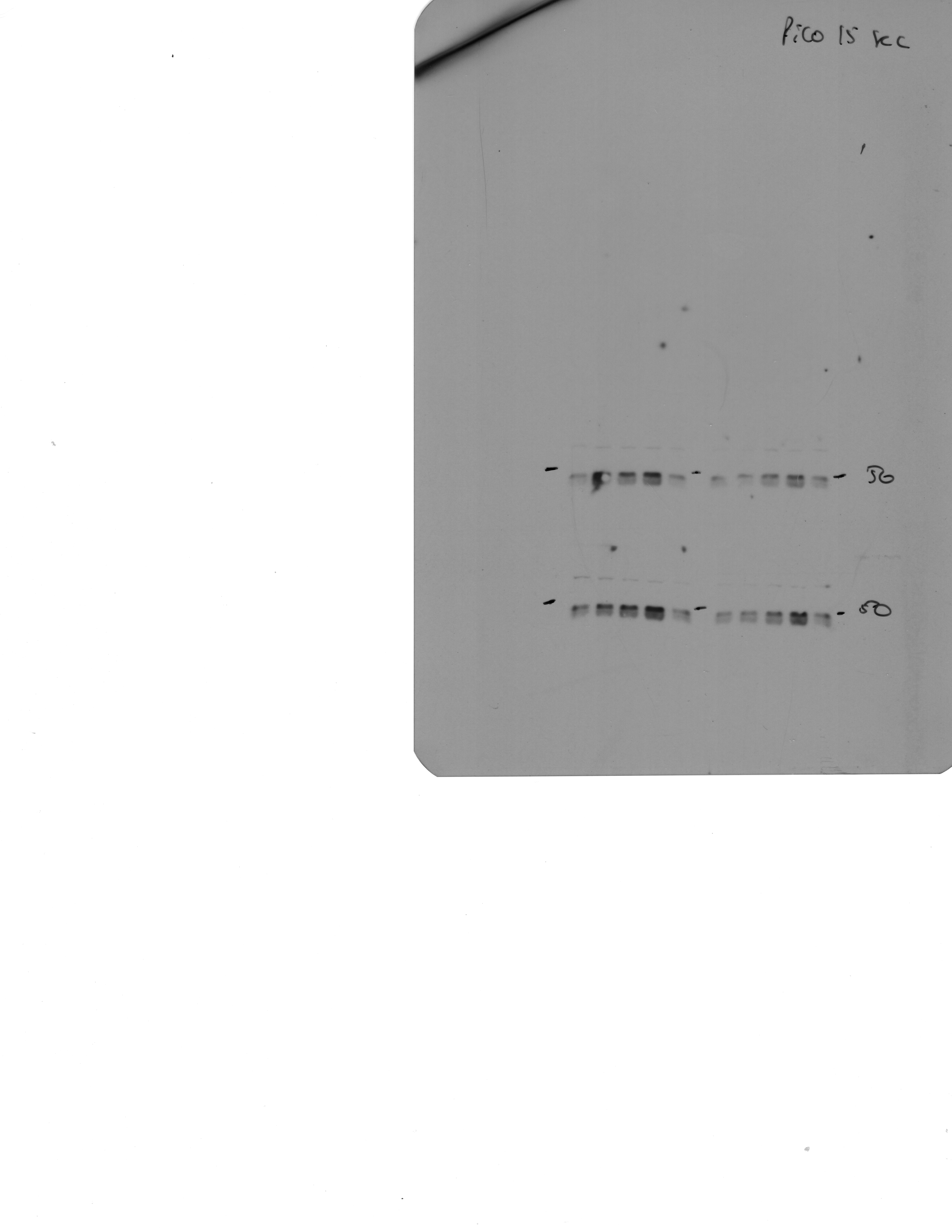

Supplement: Figure 5—figure supplement 1—source data 3. [file elife-107000-fig5-figsupp1-data3.zip › Figure 5 - figure supplement 1-source data 3/Fig S5D Total gamma2.tif]

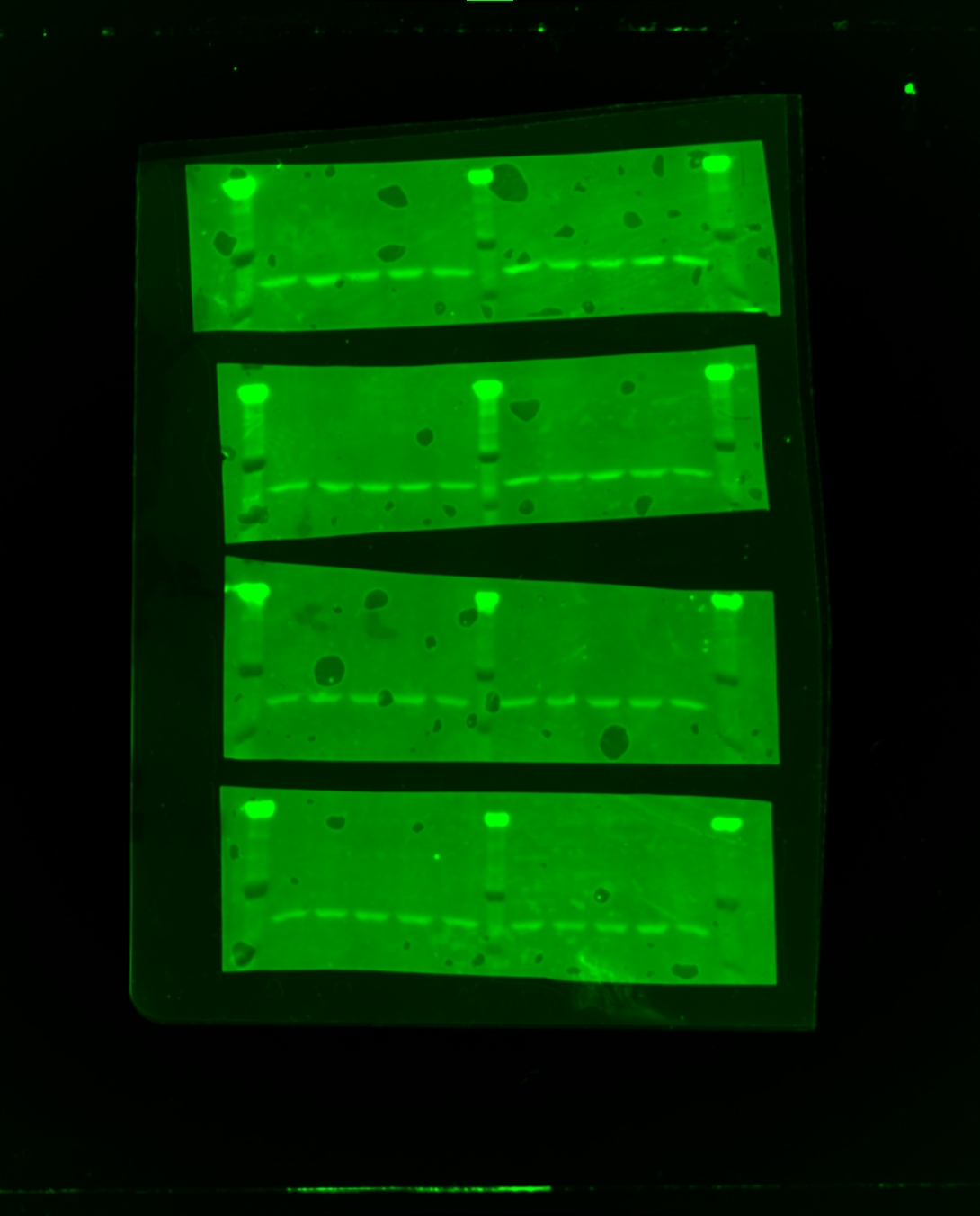

Supplement: Figure 5—figure supplement 1—source data 3. [file elife-107000-fig5-figsupp1-data3.zip › Figure 5 - figure supplement 1-source data 3/Fig S5D actin.jpg]

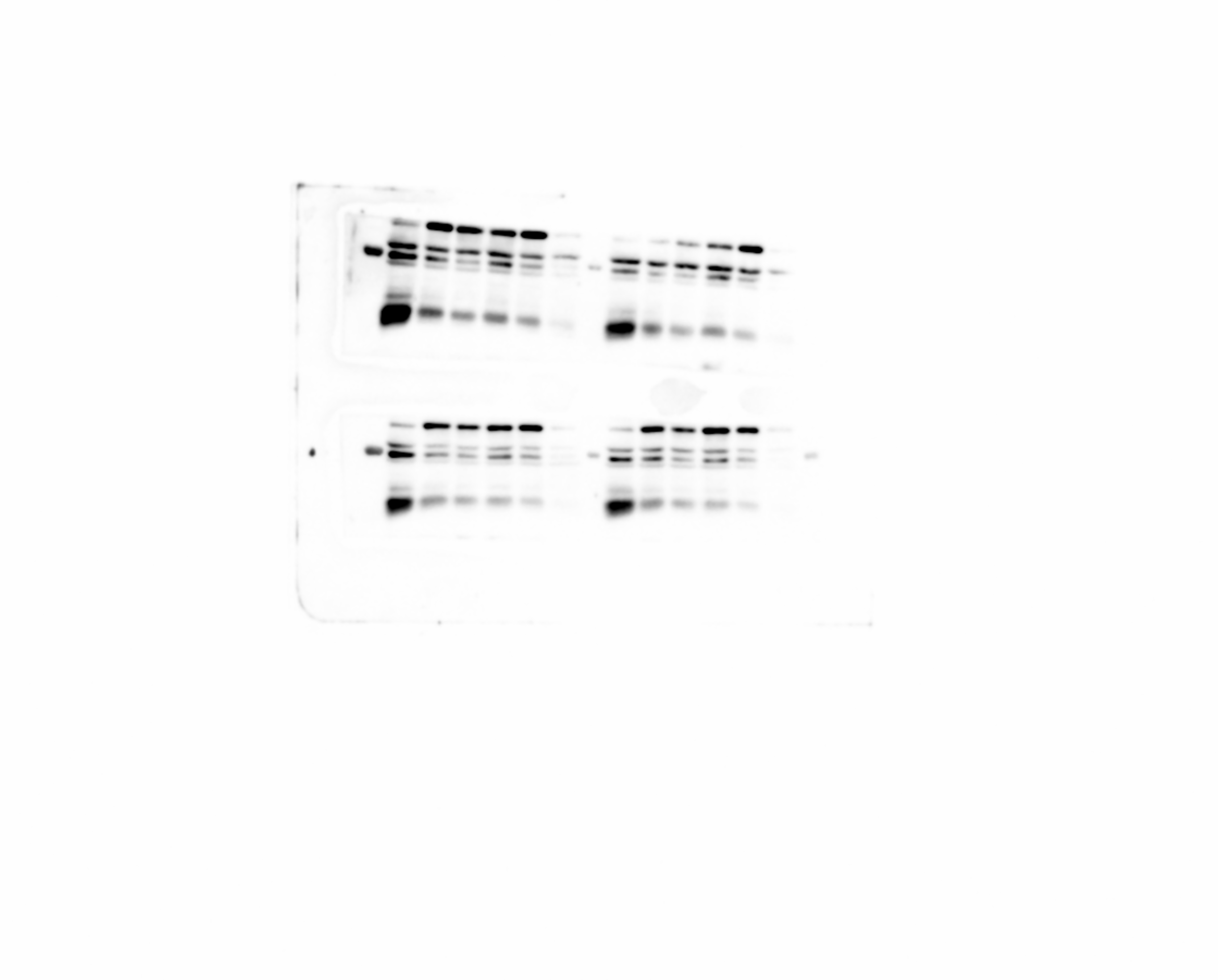

Supplement: Figure 5—figure supplement 1—source data 3. [file elife-107000-fig5-figsupp1-data3.zip › Figure 5 - figure supplement 1-source data 3/Fig S5C RG total gamma2.tif]

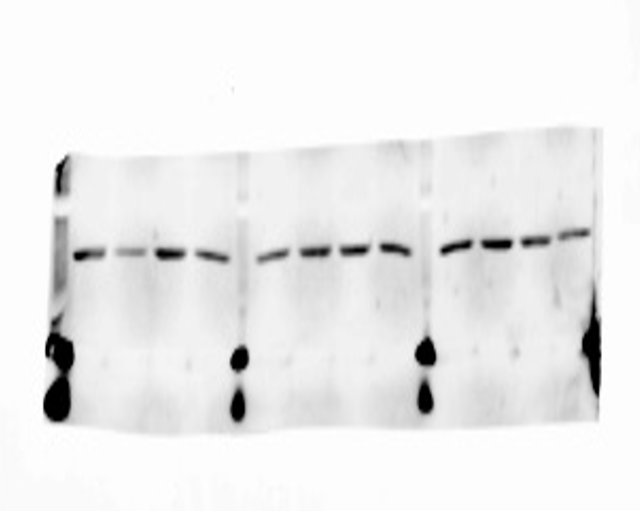

Supplement: Figure 5—figure supplement 1—source data 3. [file elife-107000-fig5-figsupp1-data3.zip › Figure 5 - figure supplement 1-source data 3/Fig S5B actin.tif]

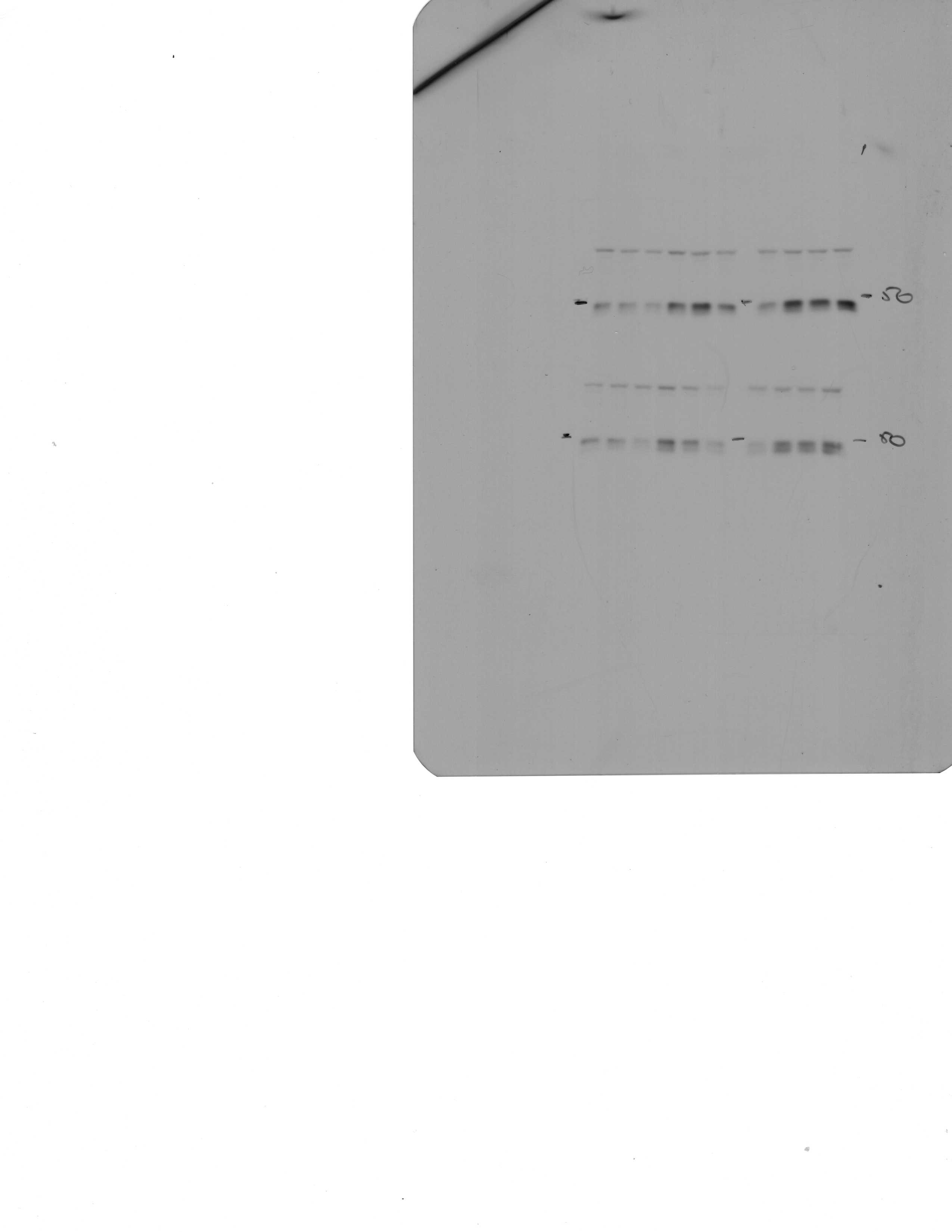

Supplement: Figure 5—figure supplement 1—source data 3. [file elife-107000-fig5-figsupp1-data3.zip › Figure 5 - figure supplement 1-source data 3/Fig S5A total gamma2.tif]
