# Supplementary material for: Phenylhydrazone-based endoplasmic reticulum proteostasis regulator compounds with enhanced biological activity
Source: eLife. 2026 Jan 26;14:RP107000. doi: 10.7554/eLife.107000 (PMC12834500; doi:10.7554/eLife.107000)

Fig S5A. Total γ2

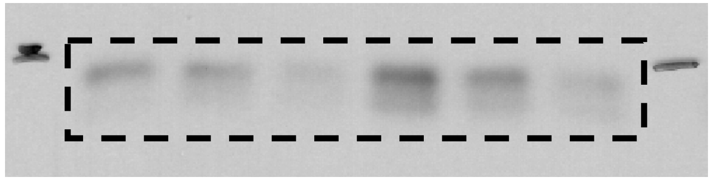

Fig S5A. β-actin

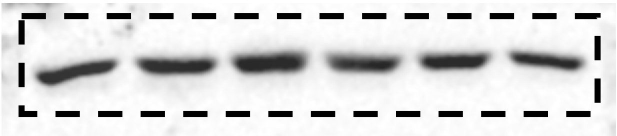

Original β-actin

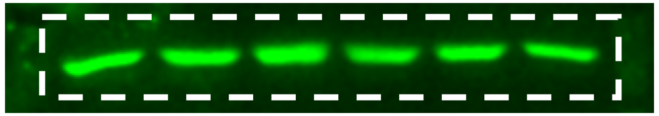

Figure S5B
IB: BiP

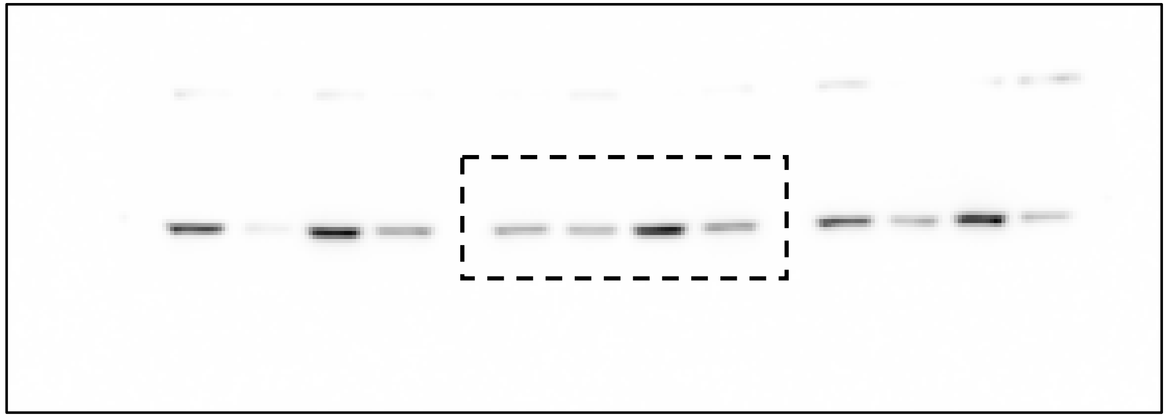

Figure S5B
IB: γ_2

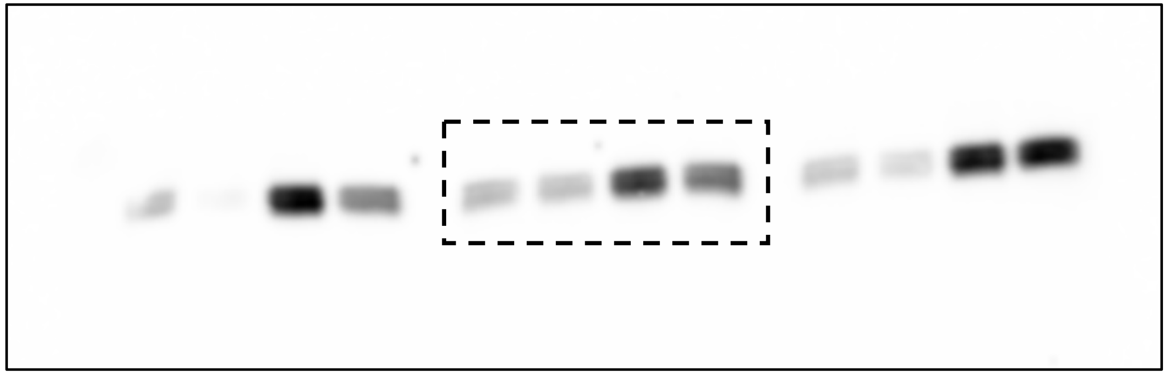

Figure S5B
IB: β-actin

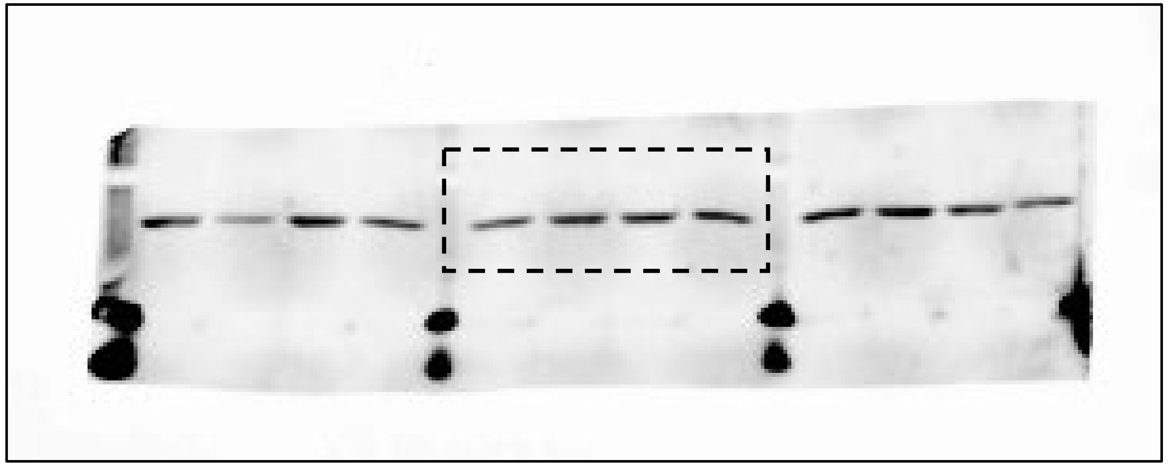

Fig S5C. α1β2γ2(WT) :
Total γ2 expression

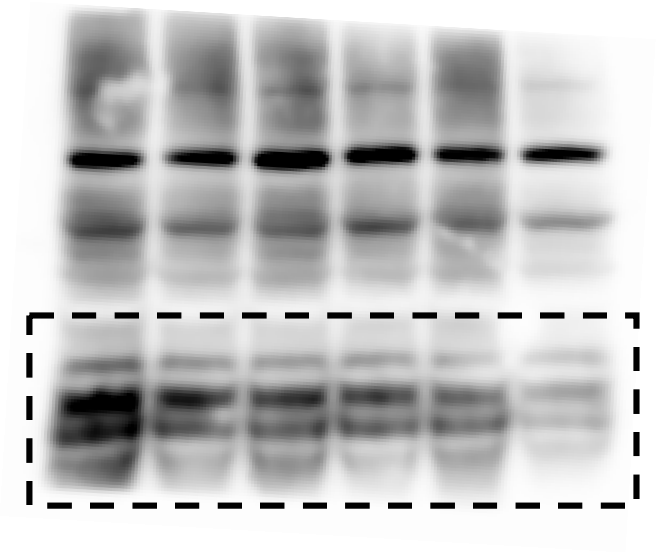

Fig S5C. α1β2γ2(R177G) :
Total γ2 expression

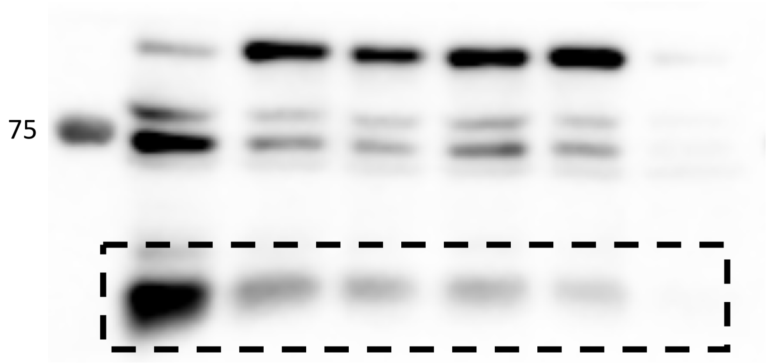

Fig S5C. α1β2γ2(R177G) + AA-263^{yne}:
Total γ2 expression

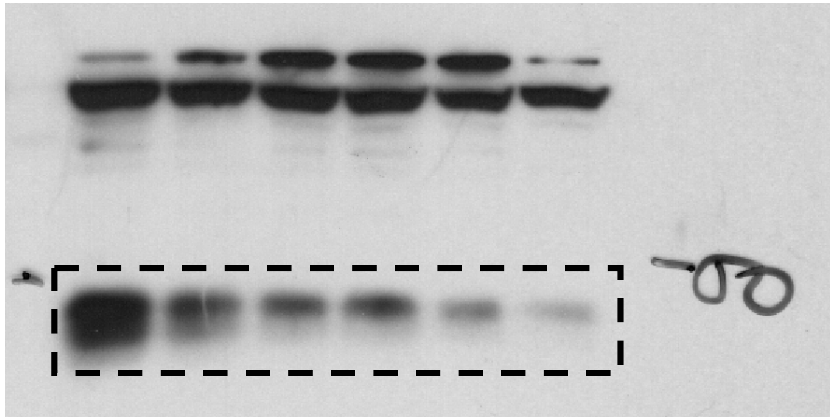

Fig S5C. α1β2γ2(WT) : β-actin

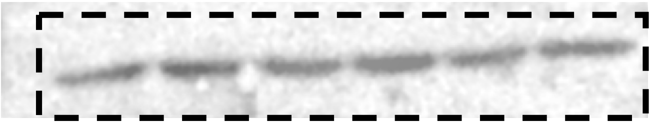

Original β-actin

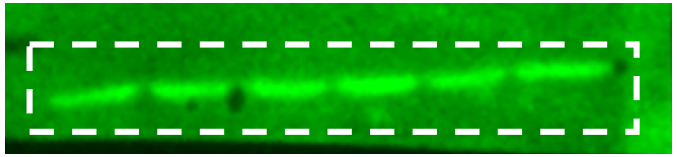

Fig S5C. α1β2γ2(R177G) : β-actin

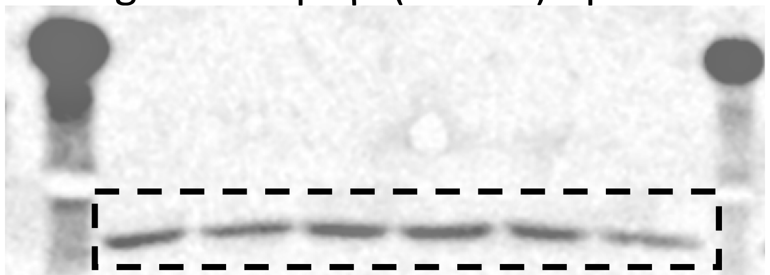

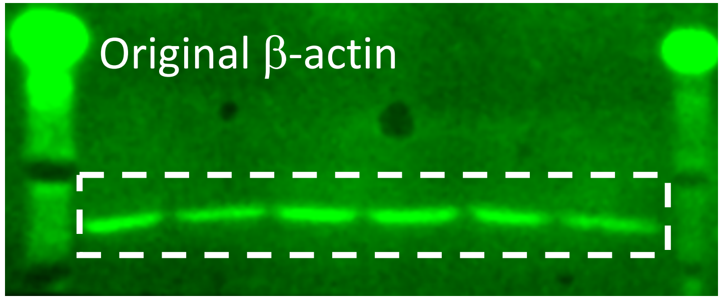

Fig S5C. α1β2γ 2(R177G) + AA-263^{yne} :
β-actin

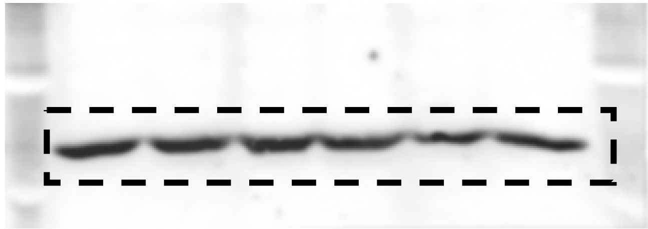

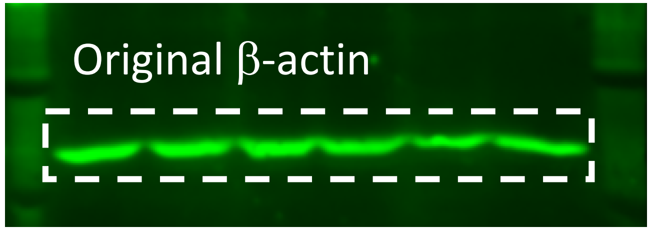

Fig S5D. Total γ2

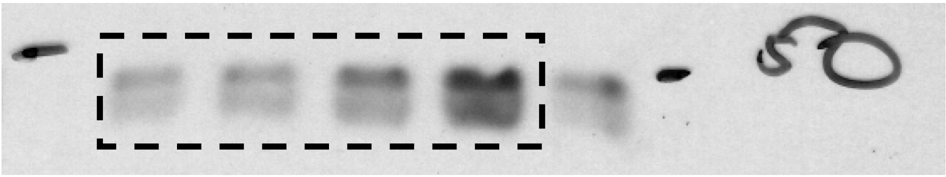

Fig S5D. β-actin

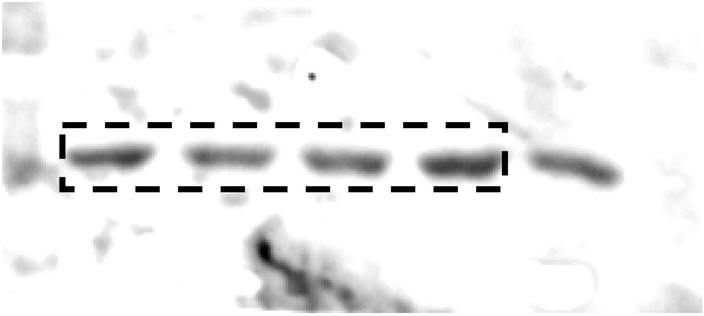

Original β-actin

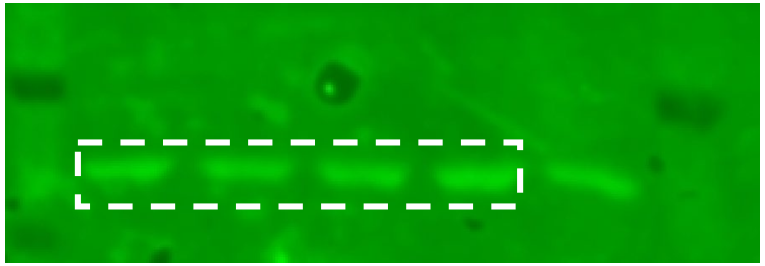

Supplement: Figure 5—figure supplement 1—source data 2. [file elife-107000-fig5-figsupp1-data2.zip › Figure 5 - figure supplement 1-source data 2/Figure 5 Figure supplement 1 source data 1.pdf]
