# Supplementary material for: Phenylhydrazone-based endoplasmic reticulum proteostasis regulator compounds with enhanced biological activity
Source: eLife. 2026 Jan 26;14:RP107000. doi: 10.7554/eLife.107000 (PMC12834500; doi:10.7554/eLife.107000)
Supplement: Supplementary file 3. [file elife-107000-supp3.pdf]

Supplemental Materials and Methods for:

Phenylhydrazone-based Endoplasmic Reticulum Proteostasis Regulator Compounds with Enhanced Biological Activity

Gabriel M. Kline[1], Lisa Boinon[2], Adrian Guerrero[1], Sergei Kutseikin[3], Gabrielle Cruz[3], Marnie P. Williams[2], Ryan J. Paxman[1], William E. Balch[3], Jeffery W. Kelly[1,*], Tingwei Mu[2,*], R. Luke Wiseman[3,*]

[1]Department of Chemistry, The Scripps Research Institute, La Jolla, CA 92037

[2]The Department of Physiology and Biophysics, Case Western Reserve University, Cleveland, OH 44106

[3]Department of Molecular and Cellular Biology, The Scripps Research Institute, La Jolla, CA 92037

*To whom correspondences should be addressed:

R. Luke Wiseman	Tingwei Mu	Jeffery W. Kelly
Email: wiseman@scripps.edu	Email: txm210@case.edu	Email: jkelly@scripps.edu
Phone: (858) 784-8820	Phone: (216)-368-0750	Phone: (858)-784-9880

Running Title: Improved ER proteostasis regulators for biological applications.

General Synthetic Procedures

All compounds and reagents were purchased from Sigma-Aldrich, Acros, Alfa Aesar, Combi-blocks, and EMD Millipore unless otherwise noted and were used without further purification. Thin layer chromatography with Merck silica plates (60-F254), using UV light as the visualizing agent, was used to monitor reaction progress. Flash column chromatography was carried out using a Teledyne Isco Combiflash Nextgen 300+ machine using Luknova SuperSep columns (SiO_2,25 μm) with ethyl acetate and hexanes as eluents. ^{1}H NMR spectra were recorded on a Varian INOVA-400 400MHz spectrometer. Chemical shifts are reported in δ units (ppm) relative to residual solvent peak. Coupling constants (*J*) are reported in hertz (Hz). Characterization data are reported as follows: chemical shift, multiplicity (s=singlet, d=doublet, t=triplet, q=quartet, br=broad, m=multiplet), coupling constants, number of protons, mass to charge ratio. The compound's identity was confirmed via high-resolution mass spectrometry.

AA263 Analog Representative Synthetic Procedure (AA263-1:AA263-12)

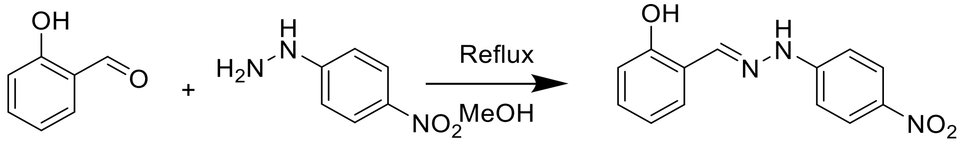

To a round bottom flask equipped with a stir bar was added salicaldehyde (1 mmol, 1 eq, 107 μL) and 4-nitrophenylhydrazine (1 mmol, 1 eq, 153.14 mg) in 5 mL MeOH. Solution refluxed until reaction complete by TLC. Solvent removed under reduced pressure and crude residue purified by column chromatography (SiO_2 4:1 Hex, EtOAc) to give product as yellow/orange powder.

AA263^{yne} Analog Representative Synthetic Procedure (AA263-13:AA263-20)

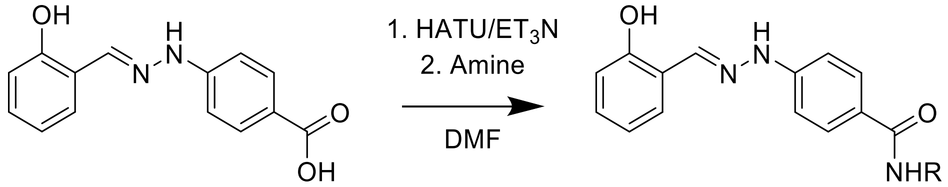

Charged scintillation vial with 263-3 (0.2 mmol, 1 eq, 31.2 mg) and HATU (0.25 mmol, 1.25 eq, 95 mg). Dissolved solids in 1 mL DMF before addition of triethylamine (1.5 eq, 0.3 mmol, 41 µL). After stirring for five minutes, amine (0.4 mmol, 2 eq) dissolved in .5 mL DMF and added dropwise. Reaction left to stir for 2h-overnight until completion, after which the reaction mixture was poured into water. Aqueous layer extracted with ethyl acetate (20 mLx3) and the combined organic layers were washed sequentially with water, 1M HCl and brine. After drying over $MgSO_4$, the crude residue purified by column chromatography (SiO_2, 1:1 Hex/EtOAc) to give product as light yellow solid.

Compound Characterization

AA263

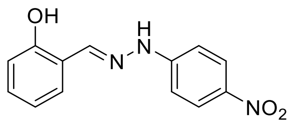

^{1}H NMR (400 MHz, DMSO) δ 11.30 (s, 1H), 10.15 (s, 1H), 8.37 (s, 1H), 8.18 – 8.11 (m, 2H), 7.74 (dd, *J* = 7.7, 1.7 Hz, 1H), 7.28 – 7.18 (m, 1H), 7.11 (d, *J* = 8.8 Hz, 2H), 6.89 (dd, *J* = 13.0, 7.6 Hz, 2H). LC-MS $[M+H]^+$: 258.1

AA263-1

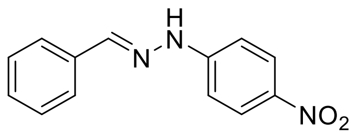

^{1}H NMR (400 MHz, Acetone-d_6) δ 10.43 (s, 1H), 8.32 (d, *J* = 9.2 Hz, 2H), 8.21 (s, 1H), 7.95 – 7.87 (m, 2H), 7.63 – 7.48 (m, 3H), 7.43 (d, *J* = 9.0 Hz, 2H). LC-MS $[M+H]^+$: 241.1

AA263^{yne}

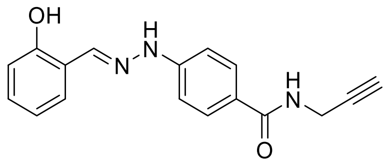

^{1}H NMR (400 MHz, DMSO-D_6) δ 10.69 (s, 1H), 10.27 (s, 1H), 8.60 (t, *J* = 5.7 Hz, 1H), 8.18 (s, 1H), 7.74 (d, *J* = 8.4 Hz, 2H), 7.60 (d, *J* = 7.7 Hz, 1H), 7.14 (t, *J* = 7.5 Hz, 1H), 6.95 (d, *J* = 8.4 Hz, 2H), 6.88 – 6.79 (m, 2H), 3.98 (dd, *J* = 5.7, 2.5 Hz, 2H), 3.06 (s, 1H). LC-MS $[M+H]^+$: 294.1

AA263-2

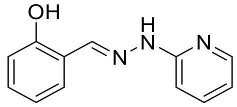

^{1}H NMR (400 MHz, DMSO-D6) δ 10.89 (s, 1H), 10.46 (s, 1H), 8.25 (s, 1H), 8.09 (dd, J = 5.0, 1.8 Hz, 1H), 7.60 (ddd, J = 8.8, 7.2, 1.9 Hz, 1H), 7.54 (dd, J = 7.7, 1.7 Hz, 1H), 7.14 (td, J = 7.7, 1.7 Hz, 1H), 7.01 (d, J = 8.4 Hz, 1H), 6.83 (dd, J = 12.9, 7.8 Hz, 2H), 6.73 (dd, J = 7.1, 4.9 Hz, 1H). LC-MS $[M+H]^+$: 214.1

AA263-3

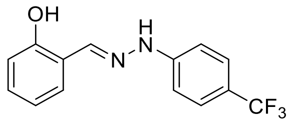

^{1}H NMR (400 MHz, DMSO-D6) δ 10.78 (s, 1H), 10.20 (s, 1H), 8.21 (s, 1H), 7.62 (dd, J = 7.8, 1.7 Hz, 1H), 7.51 (d, J = 8.5 Hz, 2H), 7.15 (ddd, J = 8.5, 7.3, 1.7 Hz, 1H), 7.07 (d, J = 8.5 Hz, 2H), 6.89 – 6.79 (m, 2H). LC-MS $[M+H]^+$: 281.1

AA263-4

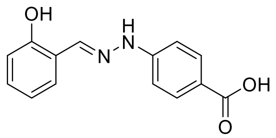

^{1}H NMR (400 MHz, DMSO-D6) δ 12.33 (s, 1H), 10.81 (s, 1H), 10.23 (s, 1H), 8.21 (s, 1H), 7.84 – 7.74 (m, 2H), 7.61 (dd, J = 7.7, 1.7 Hz, 1H), 7.15 (ddd, J = 8.5, 7.2, 1.7 Hz, 1H), 7.02 – 6.92 (m, 2H), 6.88 – 6.75 (m, 2H). LC-MS $[M+H]^+$: 257.1

AA263-5

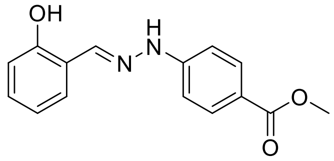

^{1}H NMR (400 MHz, DMSO-D6) δ 10.85 (s, 1H), 10.20 (s, 1H), 8.22 (s, 1H), 7.84 – 7.77 (m, 2H), 7.63 (dd, J = 7.7, 1.7 Hz, 1H), 7.15 (ddd, J = 8.5, 7.2, 1.7 Hz, 1H), 7.04 – 6.96 (m, 2H), 6.89 – 6.77 (m, 2H), 3.74 (s, 3H). LC-MS $[M+H]^+$: 271.1

AA263-7

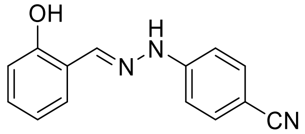

^{1}H NMR (400 MHz, DMSO-D6) δ 10.93 (s, 1H), 10.13 (d, J = 0.9 Hz, 1H), 8.23 (s, 1H), 7.65 (d, J = 7.7 Hz, 1H), 7.62 – 7.56 (m, 2H), 7.20 – 7.11 (m, 1H), 7.03 (d, J = 8.4 Hz, 2H), 6.83 (dd, J = 13.2, 7.8 Hz, 2H). LC-MS $[M+H]^+$: 238.1

AA263-8

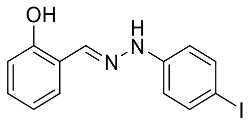

^{1}H NMR (400 MHz, DMSO-D6) δ 10.47 (s, 1H), 10.27 (s, 1H), 8.11 (s, 1H), 7.55 (dd, J = 7.7, 1.7 Hz, 1H), 7.52 – 7.44 (m, 2H), 7.13 (ddd, J = 8.7, 7.3, 1.7 Hz, 1H), 6.87 – 6.74 (m, 4H). LC-MS $[M+H]^+$: 339.0

AA263-9

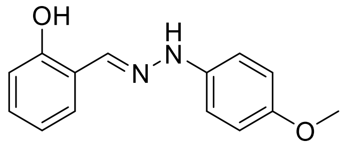

^{1}H NMR (400 MHz, DMSO-D6) δ 11.29 (s, 1H), 8.35 (s, 1H), 8.09 (dd, J = 9.4, 1.3 Hz, 2H), 7.86 (dd, J = 7.7, 1.7 Hz, 1H), 7.33 (ddd, J = 8.7, 7.4, 1.7 Hz, 1H), 7.09 (d, J = 8.7 Hz, 2H), 7.05 (dd, J = 8.5, 1.1 Hz, 1H), 6.97 (t, J = 7.5 Hz, 1H), 3.82 (s, 3H). 243.1

AA263-10

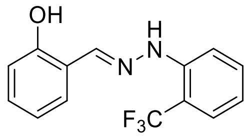

^{1}H NMR (400 MHz, DMSO-D6) δ 10.20 (s, 1H), 9.80 (s, 1H), 8.61 (s, 1H), 7.65 – 7.46 (m, 4H), 7.16 (ddd, J = 8.7, 7.2, 1.7 Hz, 1H), 6.94 – 6.79 (m, 3H). LC-MS $[M+H]^+$: 281.1

AA263-11

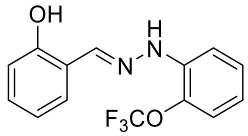

^{1}H NMR (400 MHz, DMSO-D6) δ 10.52 (s, 1H), 10.27 (s, 1H), 8.14 (s, 1H), 7.56 (dd, J = 7.7, 1.7 Hz, 1H), 7.24 – 7.16 (m, 2H), 7.13 (ddd, J = 8.6, 7.3, 1.7 Hz, 1H), 7.03 – 6.95 (m, 2H), 6.88 – 6.78 (m, 2H). LC-MS $[M+H]^+$: 297.1

AA263-12

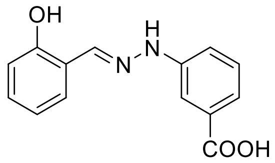

^{1}H NMR (400 MHz, DMSO-D6) δ 12.85 (s, 1H), 10.54 (s, 1H), 10.35 (s, 1H), 8.14 (s, 1H), 7.59 – 7.51 (m, 2H), 7.36 – 7.27 (m, 2H), 7.21 – 7.09 (m, 2H), 6.88 – 6.79 (m, 2H). LC-MS $[M+H]^+$: 257.1

AA263-13

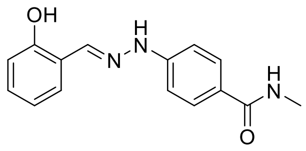

^{1}H NMR (400 MHz, DMSO) δ 10.68 (s, 1H), 10.33 (d, J = 5.2 Hz, 1H), 8.21 (s, 1H), 8.15 (s, 1H), 7.75 (s, 2H), 7.63 (s, 1H), 7.18 (s, 1H), 6.98 (s, 2H), 6.88 (s, 2H), 2.75 (s, 3H). LC-MS $[M+H]^+$: 270.1

AA263-14

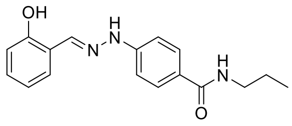

^{1}H NMR (400 MHz, METHANOL-D4) δ 7.77 – 7.72 (m, 2H), 7.65 – 7.61 (m, 2H), 7.55 – 7.51 (m, 1H), 7.30 – 7.23 (m, 1H), 7.18 (ddt, J = 8.2, 7.2, 1.4 Hz, 1H), 7.00 – 6.92 (m, 2H), 6.93 – 6.84 (m, 2H), 1.62 (p, J = 7.3 Hz, 2H), 1.33 (ddd, J = 7.5, 5.5, 2.2 Hz, 2H), 0.95 (td, J = 7.4, 0.9 Hz, 3H). $[M+H]^+$: 298.1

AA263-15

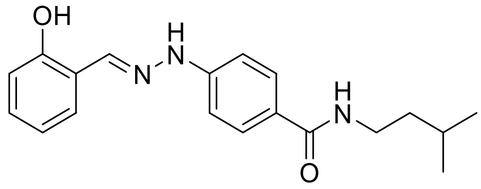

^{1}H NMR (400 MHz, DMSO) δ 10.65 (s, 1H), 10.33 (s, 1H), 8.21 (s, 1H), 8.10 (s, 1H), 7.76 (d, J = 8.3 Hz, 2H), 7.67 – 7.59 (m, 2H), 7.56 (d, J = 6.9 Hz, 1H), 7.30 – 7.24 (m, 4H), 6.98 (d, J = 8.4 Hz, 2H), 6.91 – 6.84 (m, 2H), 2.95 (s, 2H), 1.66 – 1.57 (m, 1H), 1.42 (q, J = 7.1 Hz, 2H), 0.91 (d, J = 6.6 Hz, 6H). $[M+H]^+$: 326.3

AA263-16

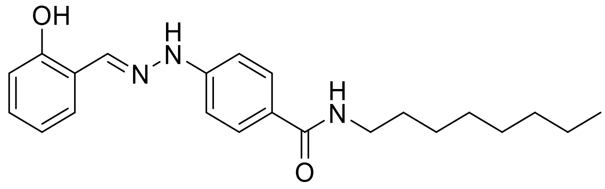

^{1}H NMR (400 MHz, DMSO) δ 10.67 (s, 1H), 10.34 (s, 1H), 8.76 (dd, J = 4.5, 1.4 Hz, 1H), 8.53 (dd, J = 8.4, 1.4 Hz, 1H), 8.21 (s, 1H), 8.15 (t, J = 5.6 Hz, 1H), 7.76 (d, J = 8.4 Hz, 2H), 7.62 (dd, J = 7.7, 1.7 Hz, 1H), 7.52 (dd, J = 8.4, 4.4 Hz, 1H), 7.23 – 7.14 (m, 1H), 6.98 (d, J = 8.4 Hz, 2H),

6.93 – 6.84 (m, 2H), 3.22 (q, J = 6.7 Hz, 2H), 1.50 (s, 2H), 1.27 (d, J = 10.3 Hz, 10H), 0.89 – 0.84 (m, 3H). $[M+H]^+$: 368.2

AA263-17

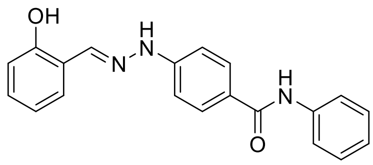

^{1}H NMR (400 MHz, DMSO) δ 10.79 (s, 1H), 10.31 (s, 1H), 9.95 (s, 1H), 8.25 (s, 1H), 7.92 (d, J = 8.5 Hz, 2H), 7.77 (d, J = 8.1 Hz, 3H), 7.65 (d, J = 7.6 Hz, 1H), 7.33 (t, J = 7.7 Hz, 3H), 7.24 – 7.16 (m, 2H), 7.10 – 7.03 (m, 4H), 6.89 (t, J = 8.9 Hz, 3H). $[M+H]^+$: 332.1

AA263-18

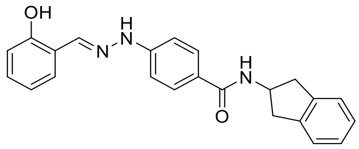

^{1}H NMR (400 MHz, DMSO) δ 10.68 (s, 1H), 10.32 (s, 1H), 8.45 (d, J = 8.4 Hz, 1H), 8.22 (s, 1H), 7.86 (d, J = 8.7 Hz, 2H), 7.62 (dd, J = 7.7, 1.7 Hz, 1H), 7.30 – 7.14 (m, 7H), 7.00 (d, J = 8.6 Hz, 2H), 6.93 – 6.84 (m, 2H), 5.56 (q, J = 8.1 Hz, 1H), 3.05 – 2.95 (m, 1H), 2.84 (dt, J = 25.9, 8.9 Hz, 2H), 2.44 (dtd, J = 11.1, 7.7, 3.0 Hz, 1H). $[M+H]^+$: 372.2

AA263-19

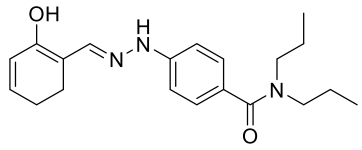

^{1}H NMR (400 MHz, DMSO) δ 10.60 (s, 1H), 10.36 (s, 1H), 8.19 (s, 1H), 7.60 (dd, J = 7.7, 1.7 Hz, 1H), 7.29 – 7.23 (m, 2H), 7.18 (ddd, J = 8.6, 7.2, 1.7 Hz, 1H), 7.03 – 6.95 (m, 2H), 6.95 – 6.83 (m, 2H), 2.69 (s, 2H), 1.54 (d, J = 8.2 Hz, 4H), 0.80 (s, 6H). $[M+H]^+$: 340.2

AA263-20

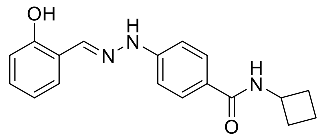

^{1}H NMR (400 MHz, DMSO) δ 10.68 (s, 1H), 10.33 (d, J = 1.4 Hz, 1H), 8.31 (d, J = 7.7 Hz, 1H), 8.22 (s, 1H), 7.81 – 7.74 (m, 2H), 7.66 – 7.59 (m, 1H), 7.23 – 7.14 (m, 1H), 7.02 – 6.95 (m, 2H), 6.93 – 6.84 (m, 2H), 4.41 (h, J = 8.2 Hz, 1H), 2.20 (d, J = 8.7 Hz, 2H), 2.13 – 1.97 (m, 2H), 1.67 (s, 2H). $[M+H]^+$: 310.1

Spectra

AA263

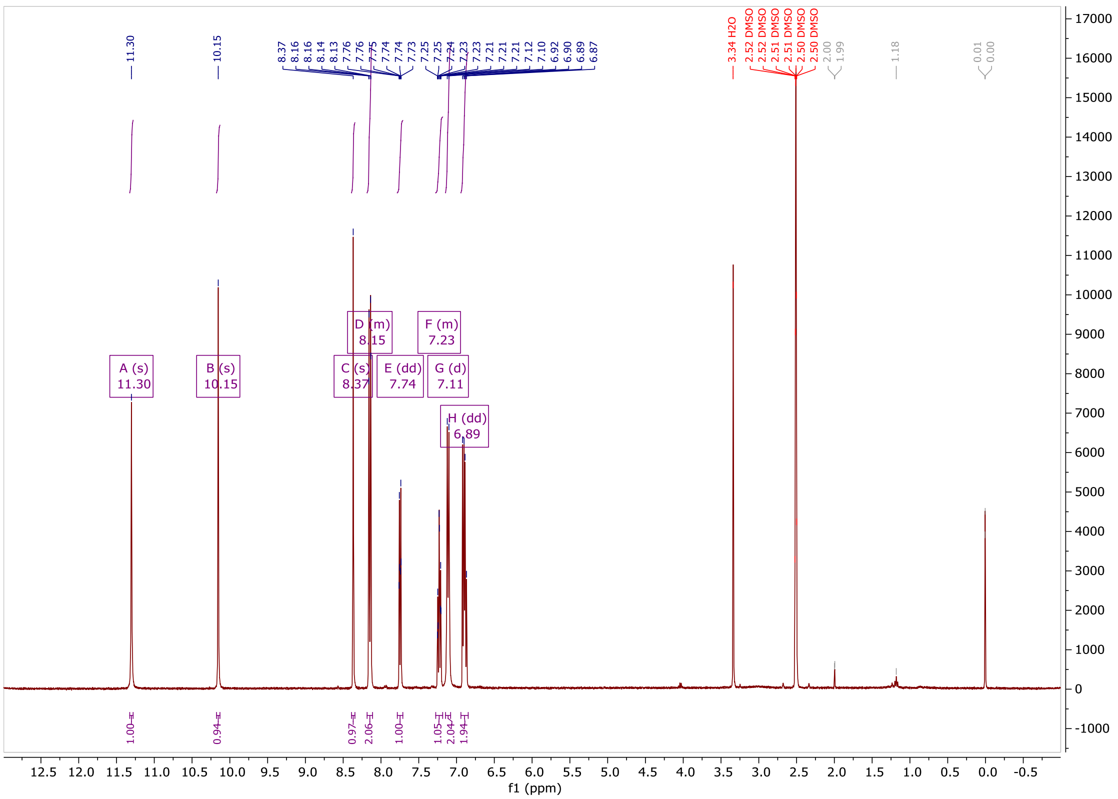

AA263-1

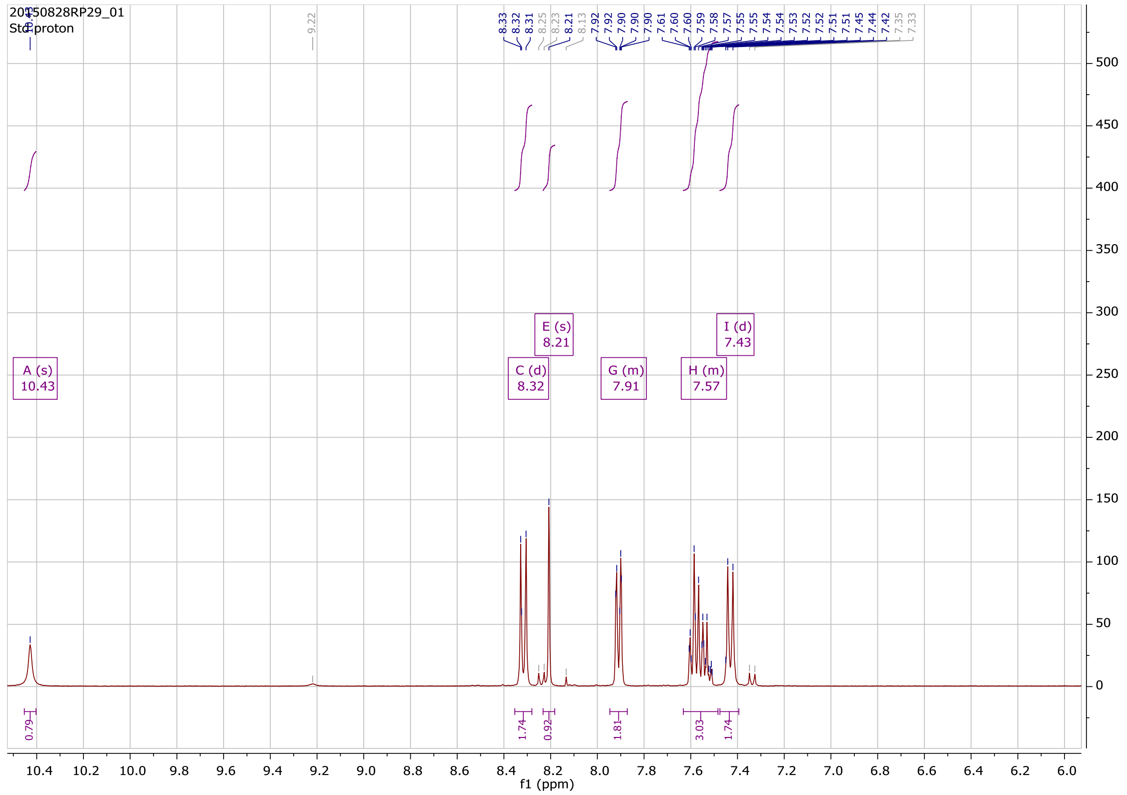

20150828RP29_01
Std proton
A (s) 10.43
C (d) 8.32
E (s) 8.21
G (m) 7.91
H (m) 7.57
I (d) 7.43
0.79
1.74
0.92
1.81
3.03
1.74
f1 (ppm)

AA263^{yne}

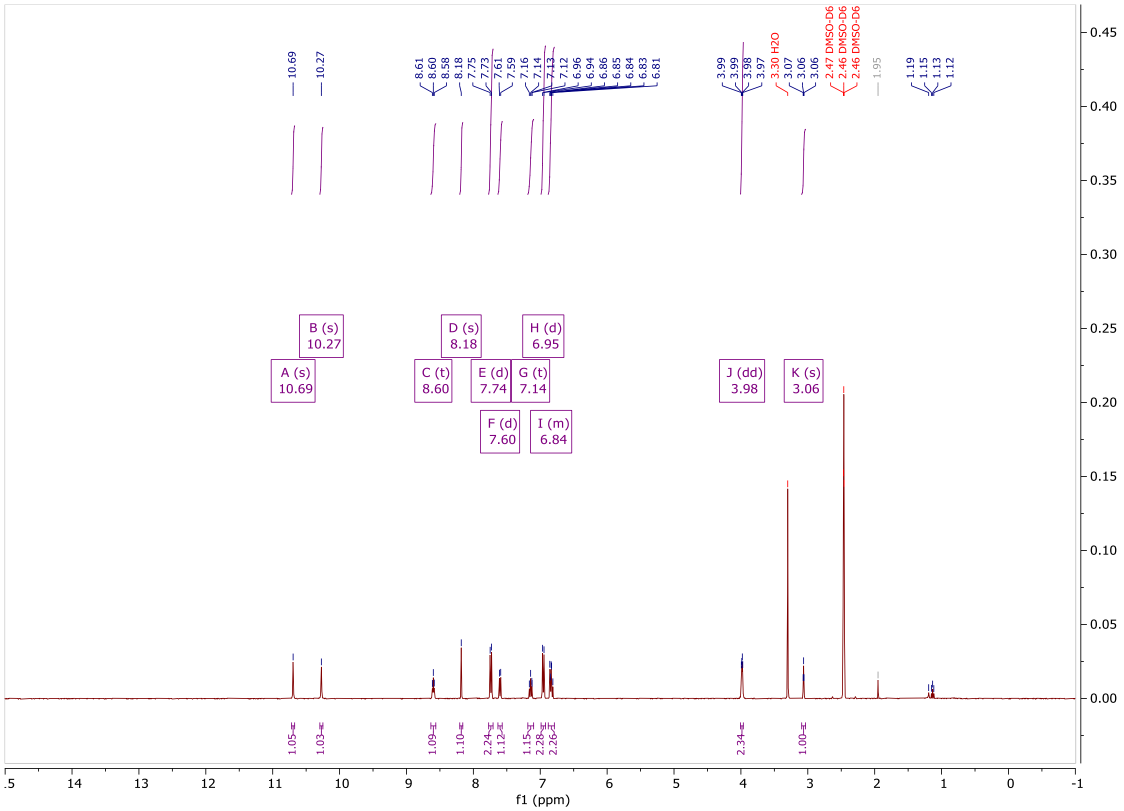
10.69
10.27
8.61
8.60
8.58
8.18
7.75
7.73
7.61
7.59
7.16
7.14
7.13
7.12
6.96
6.94
6.86
6.85
6.84
6.83
6.81
3.99
3.99
3.98
3.97
3.30 H2O
3.07
3.06
3.06
2.47 DMSO-D6
2.46 DMSO-D6
2.46 DMSO-D6
1.95
1.19
1.15
1.13
1.12
A (s) 10.69
B (s) 10.27
C (t) 8.60
D (s) 8.18
E (d) 7.74
F (d) 7.60
G (t) 7.14
H (d) 6.95
I (m) 6.84
J (dd) 3.98
K (s) 3.06
1.05
1.03
1.09
1.10
2.24
1.12
1.15
2.28
2.26
2.34
1.00
f1 (ppm)

AA263-2

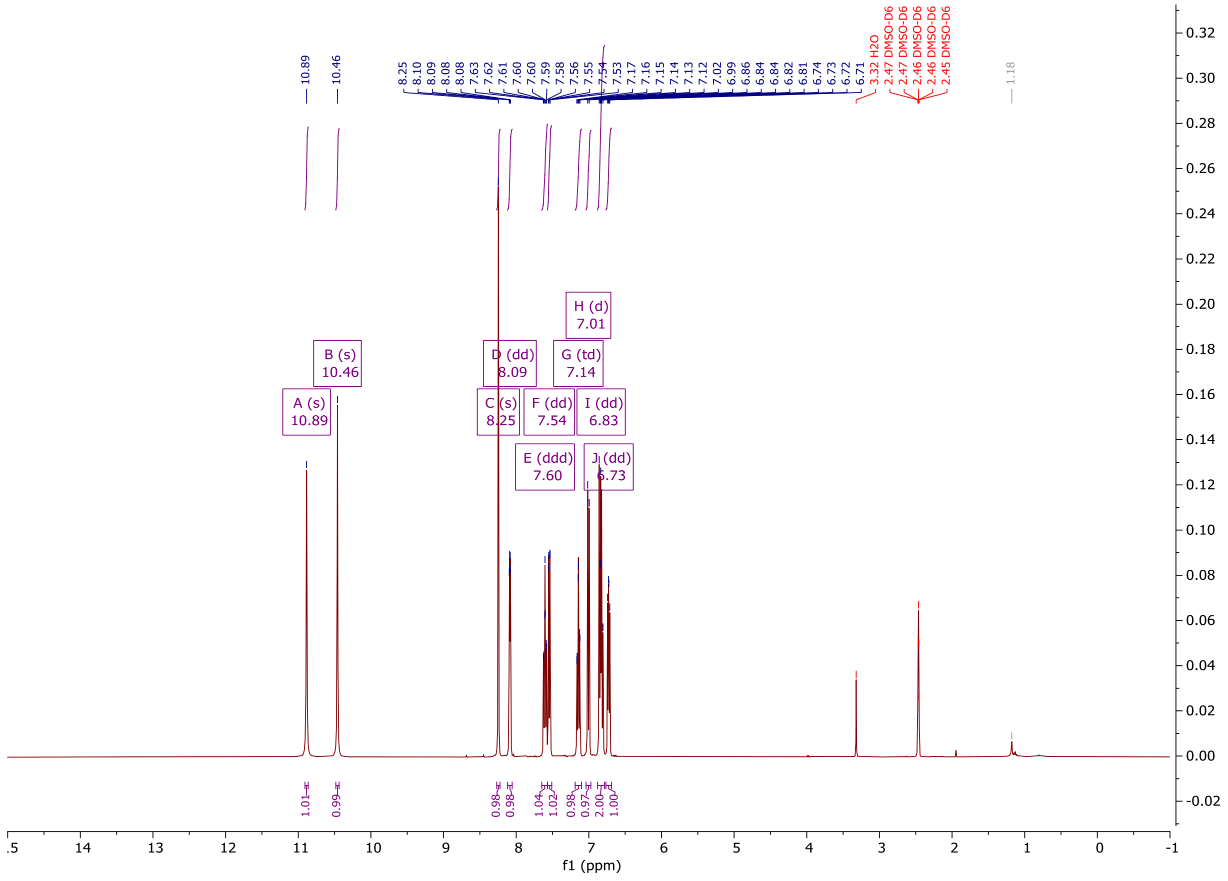

A (s) 10.89
B (s) 10.46
C (s) 8.25
D (dd) 8.09
E (ddd) 7.60
F (dd) 7.54
G (td) 7.14
H (d) 7.01
I (dd) 6.83
J (dd) 6.73
3.32 H2O
2.47 DMSO-D6
2.46 DMSO-D6
2.46 DMSO-D6
2.45 DMSO-D6
f1 (ppm)

AA263-3

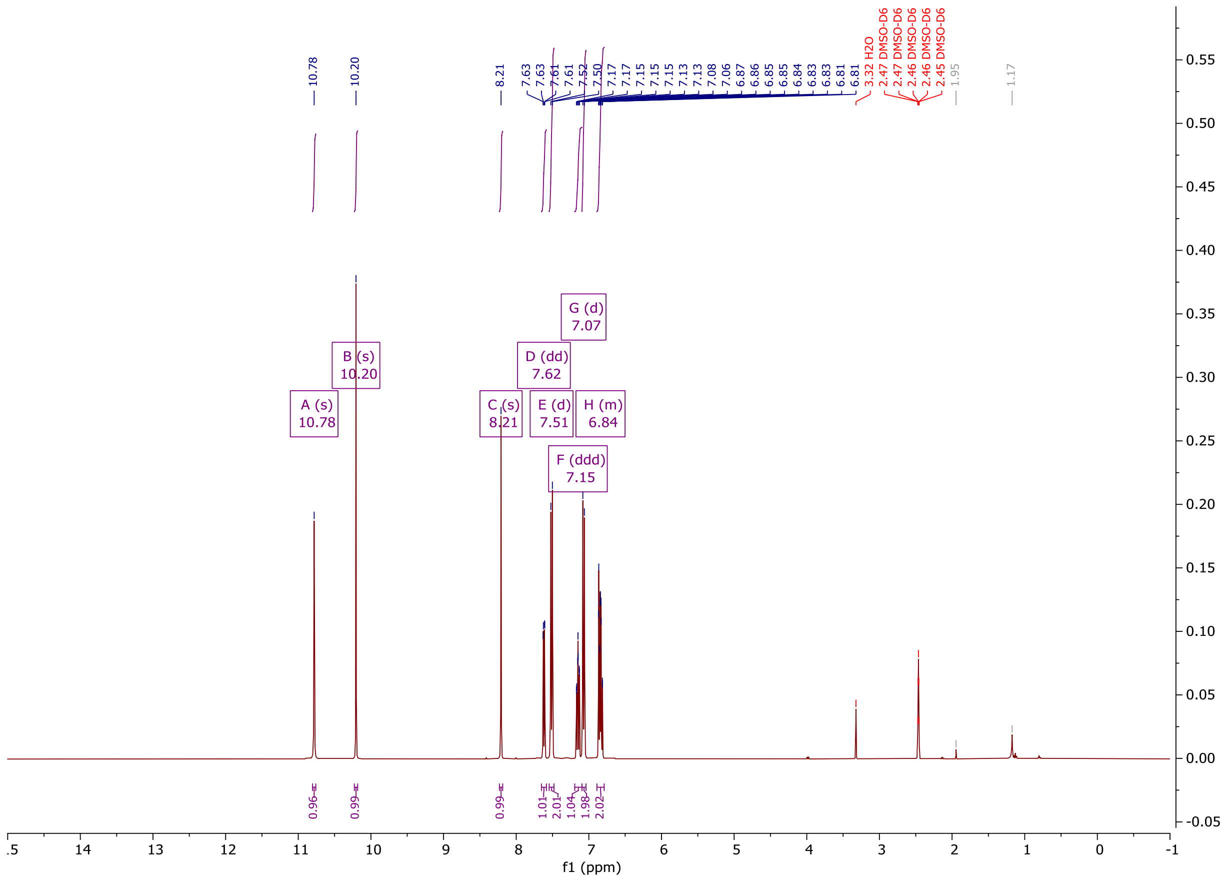

10.78
10.20
8.21
7.63
7.63
7.61
7.61
7.52
7.50
7.17
7.17
7.15
7.15
7.15
7.13
7.08
7.06
6.87
6.86
6.85
6.85
6.84
6.83
6.83
6.81
3.32 H2O
2.47 DMSO-D6
2.47 DMSO-D6
2.46 DMSO-D6
2.46 DMSO-D6
2.45 DMSO-D6
1.95
1.17
A (s) 10.78
B (s) 10.20
C (s) 8.21
D (dd) 7.62
E (d) 7.51
F (ddd) 7.15
G (d) 7.07
H (m) 6.84
0.96
0.99
0.99
1.01
2.01
1.04
1.98
2.02
f1 (ppm)

AA263-4

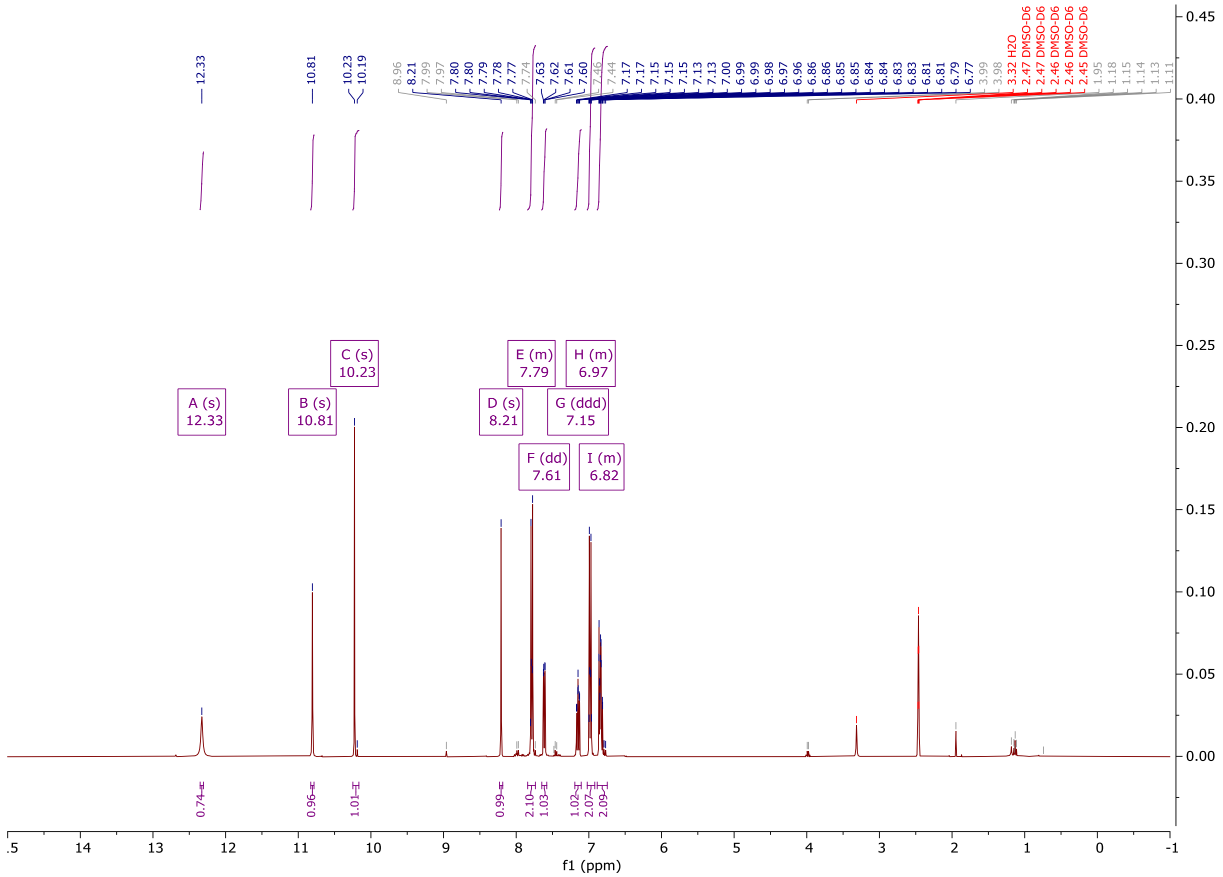
A (s)
12.33
B (s)
10.81
C (s)
10.23
D (s)
8.21
E (m)
7.79
F (dd)
7.61
G (ddd)
7.15
H (m)
6.97
I (m)
6.82
3.32 H2O
2.47 DMSO-D6
2.46 DMSO-D6
2.45 DMSO-D6
f1 (ppm)

AA263-5

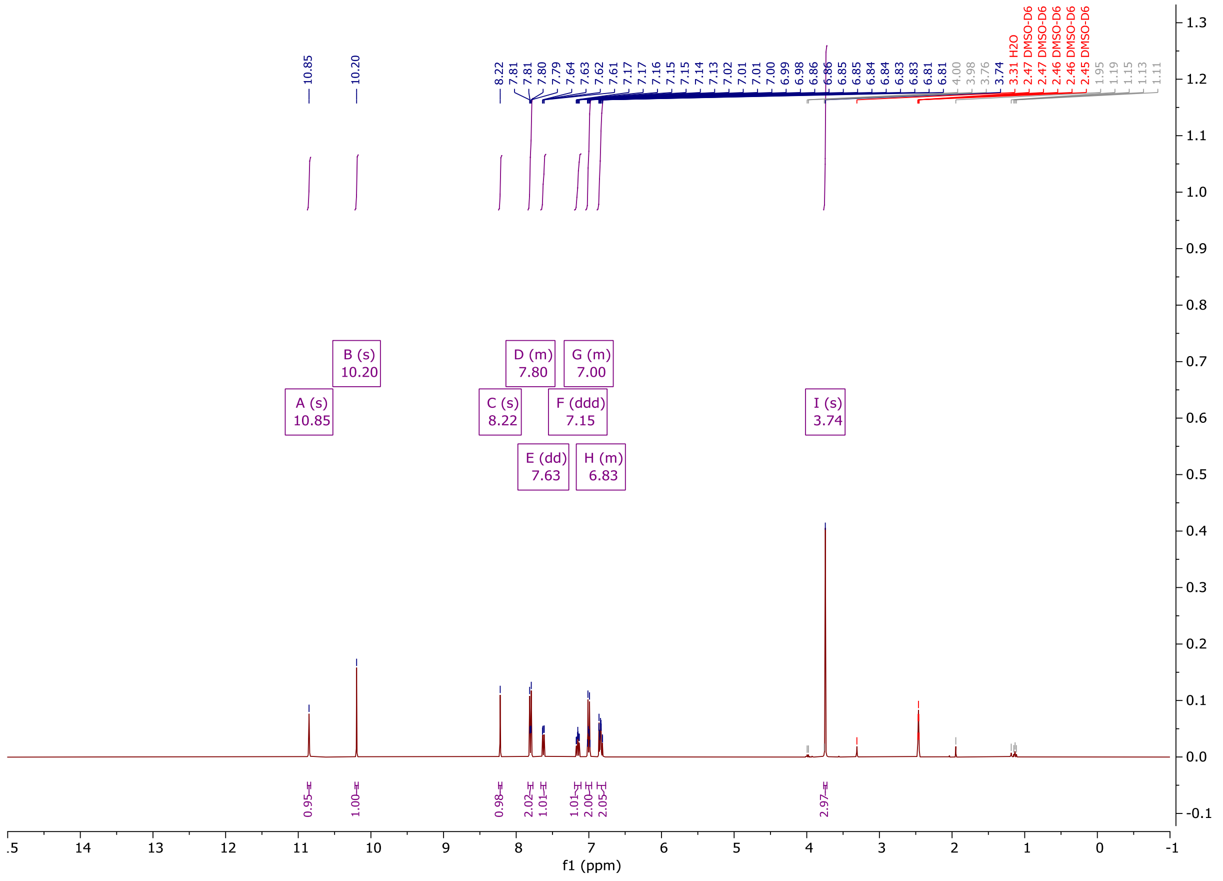

AA263-7

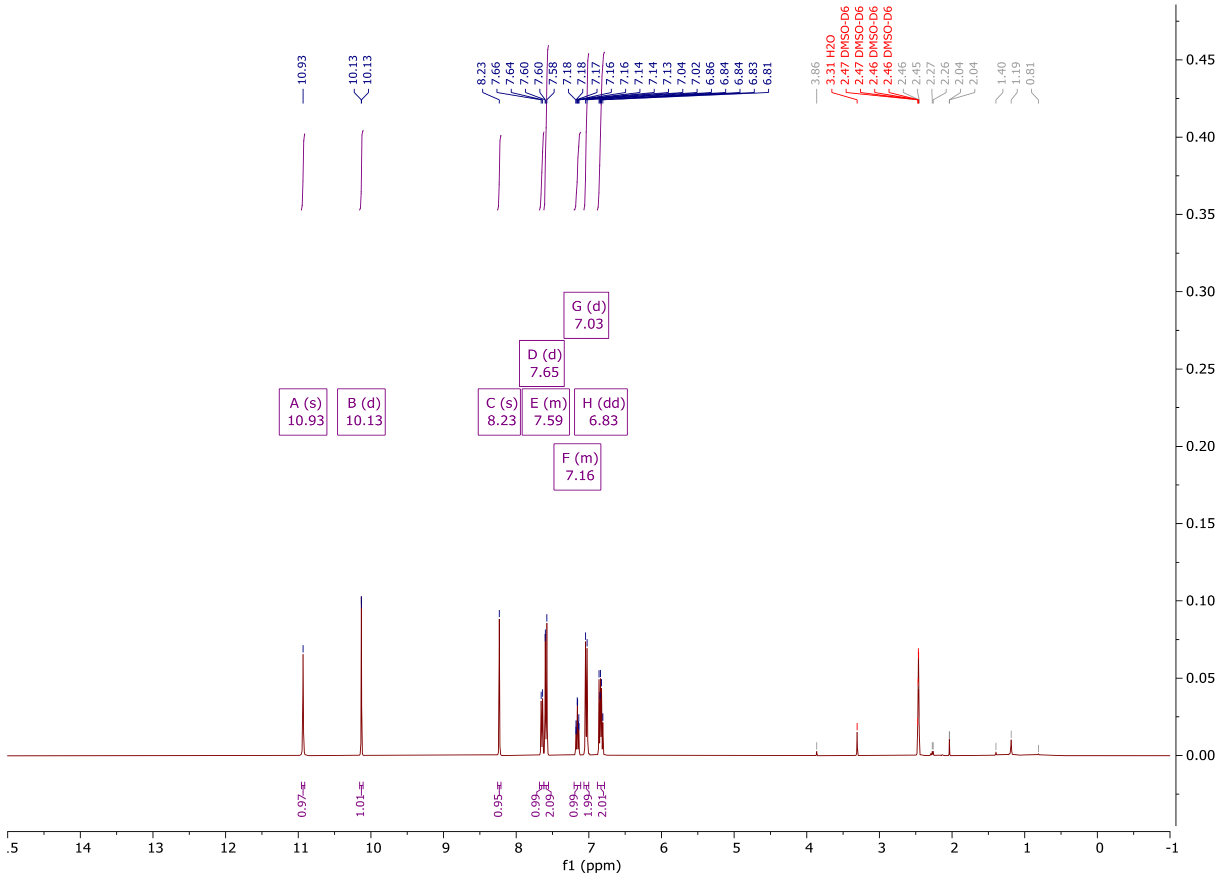

10.93
10.13
10.13
8.23
7.66
7.64
7.60
7.60
7.58
7.18
7.18
7.17
7.16
7.16
7.14
7.14
7.13
7.04
7.02
6.86
6.84
6.83
6.81
3.86
3.31 H2O
2.47 DMSO-D6
2.47 DMSO-D6
2.46 DMSO-D6
2.46 DMSO-D6
2.46
2.45
2.27
2.26
2.04
2.04
1.40
1.19
0.81
G (d) 7.03
D (d) 7.65
A (s) 10.93
B (d) 10.13
C (s) 8.23
E (m) 7.59
H (dd) 6.83
F (m) 7.16
0.97
1.01
0.95
0.99
2.09
0.99
1.99
2.01
f1 (ppm)

AA263-8

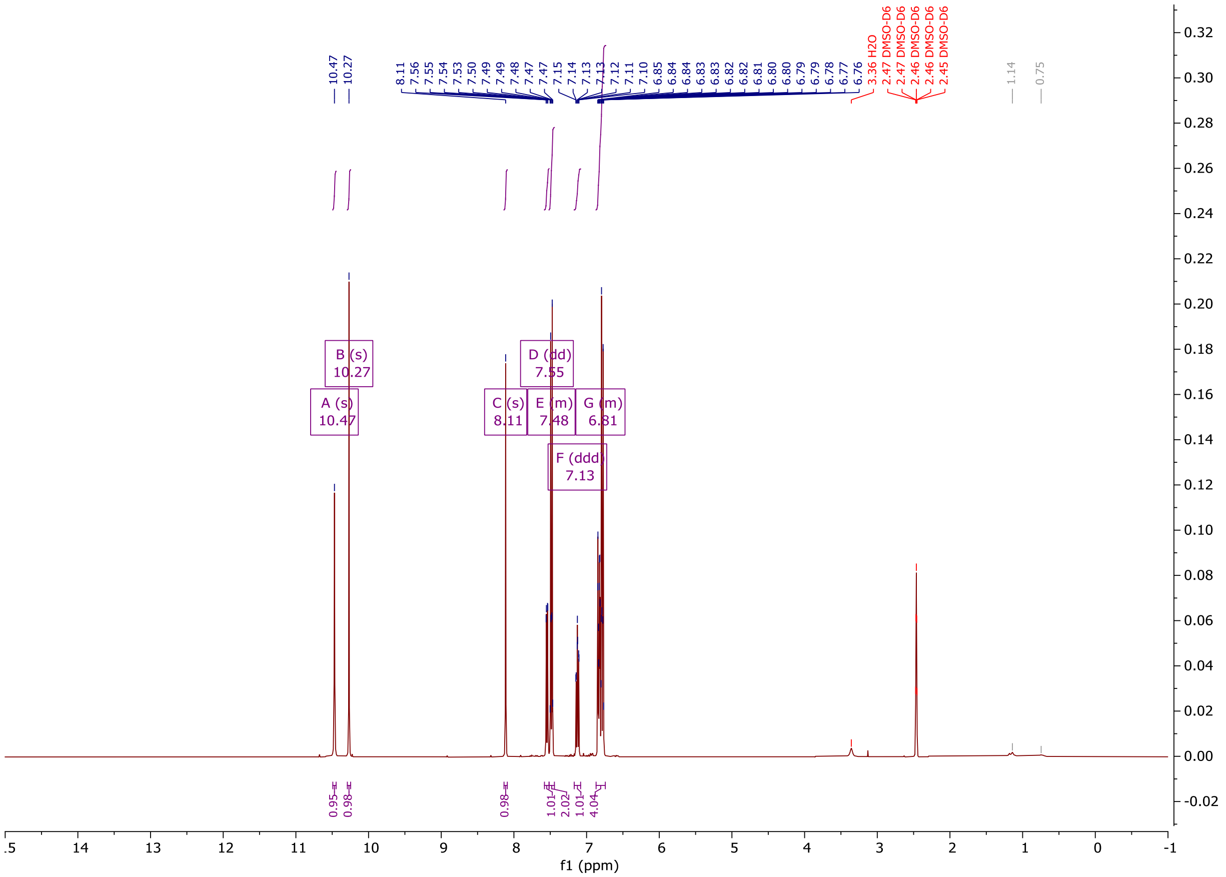

10.47
10.27
8.11
7.56
7.55
7.54
7.53
7.50
7.49
7.49
7.48
7.47
7.47
7.15
7.14
7.13
7.13
7.12
7.11
7.10
6.85
6.84
6.84
6.83
6.83
6.82
6.82
6.81
6.80
6.80
6.79
6.79
6.78
6.77
6.76
3.36 H2O
2.47 DMSO-D6
2.47 DMSO-D6
2.46 DMSO-D6
2.46 DMSO-D6
2.45 DMSO-D6
1.14
0.75
A (s) 10.47
B (s) 10.27
C (s) 8.11
D (dd) 7.55
E (m) 7.48
F (ddd) 7.13
G (m) 6.81
0.95
0.98
0.98
1.01
2.02
1.01
4.04
f1 (ppm)

AA263-9

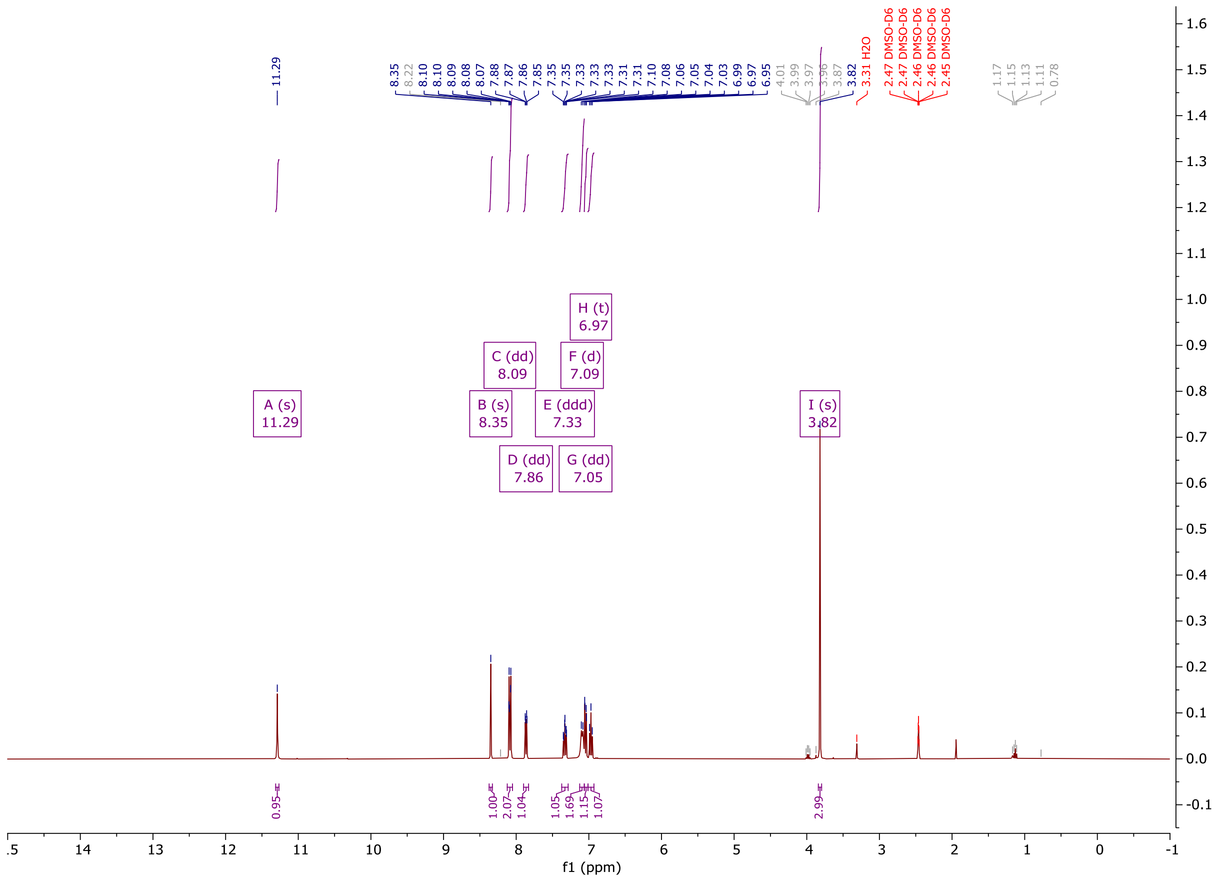
A (s)
11.29
B (s)
8.35
C (dd)
8.09
D (dd)
7.86
E (ddd)
7.33
F (d)
7.09
G (dd)
7.05
H (t)
6.97
I (s)
3.82
3.31 H2O
2.47 DMSO-D6
2.47 DMSO-D6
2.46 DMSO-D6
2.46 DMSO-D6
2.45 DMSO-D6
f1 (ppm)

AA263-10

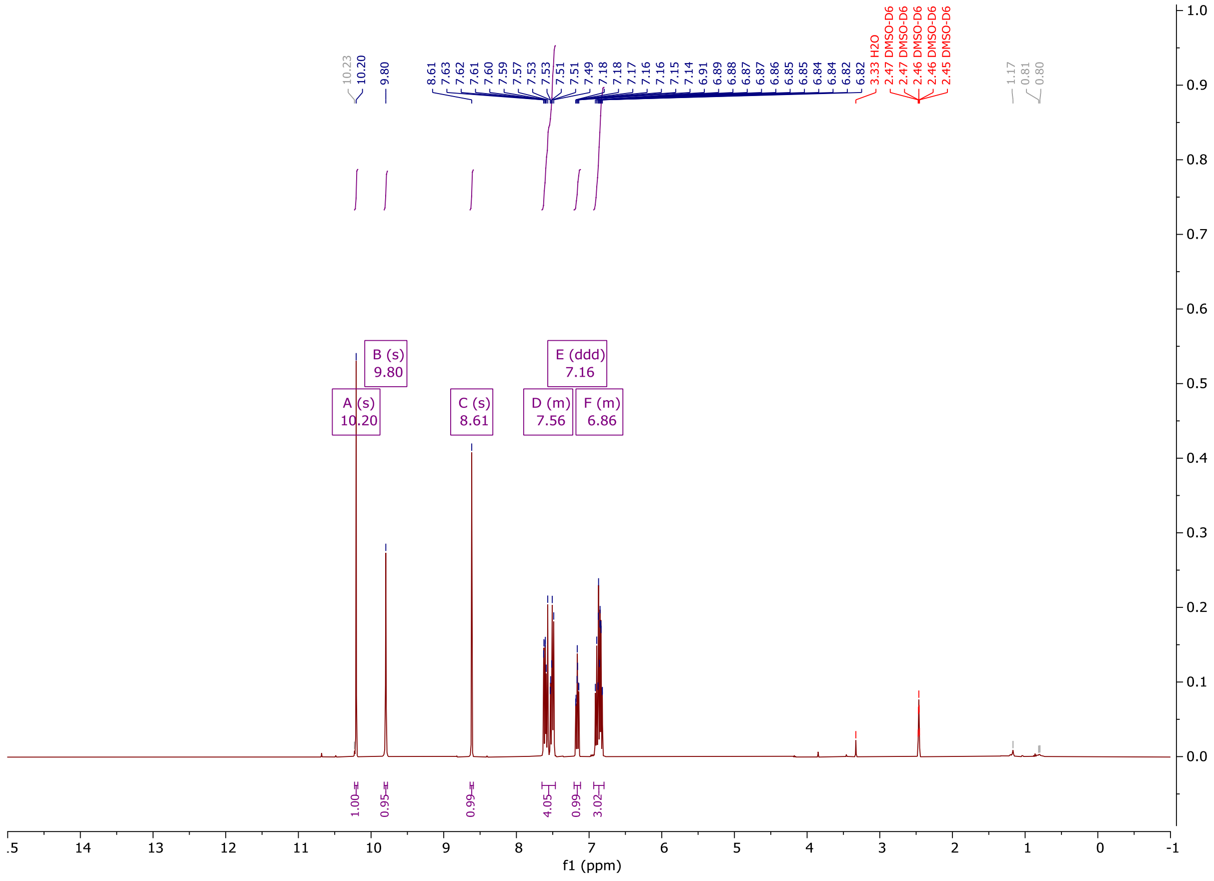

A (s)
10.20
B (s)
9.80
C (s)
8.61
D (m)
7.56
E (ddd)
7.16
F (m)
6.86
3.33 H2O
2.47 DMSO-D6
2.46 DMSO-D6
2.45 DMSO-D6
f1 (ppm)

AA263-11

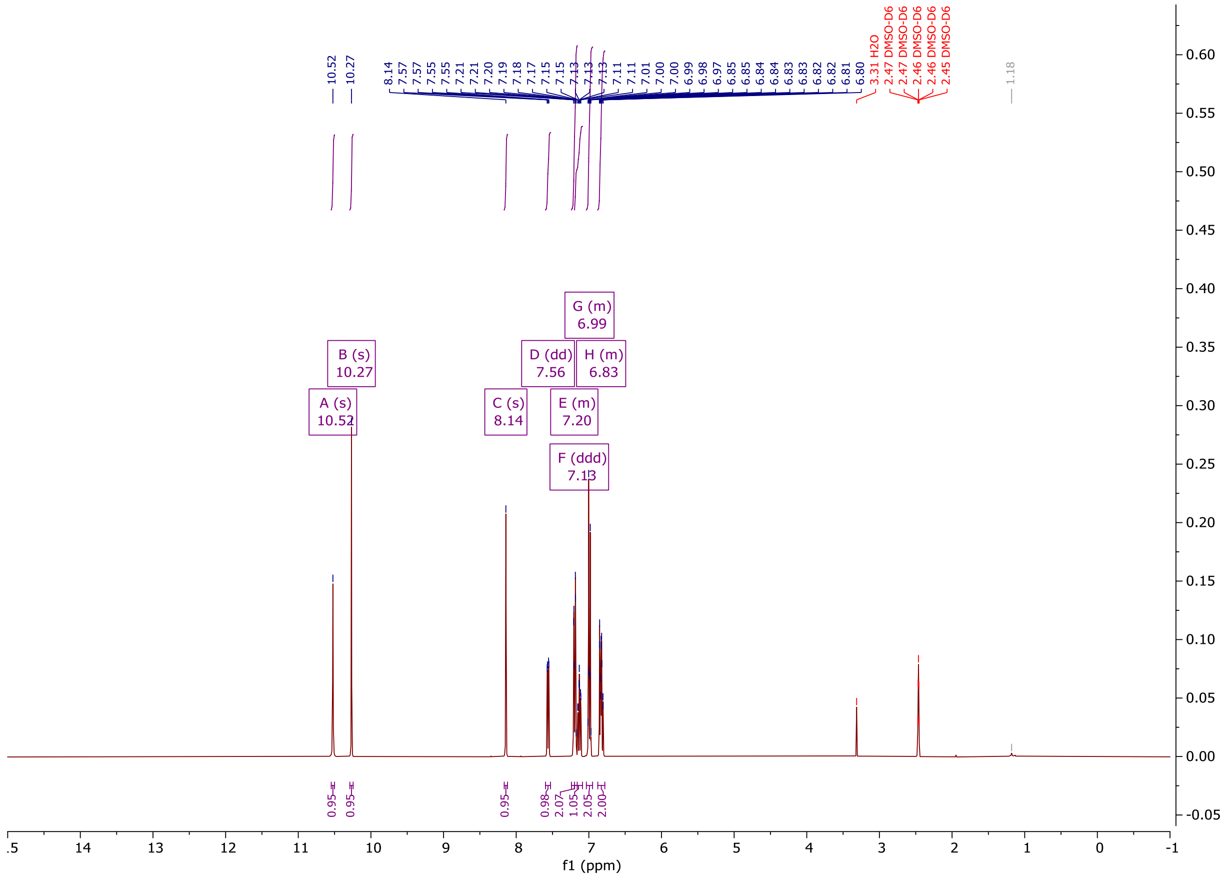

10.52
10.27
8.14 7.57 7.57 7.55 7.55 7.21 7.21 7.20 7.19 7.18 7.17 7.15 7.13 7.13 7.13 7.11 7.01 7.00 6.99 6.98 6.97 6.85 6.85 6.84 6.84 6.83 6.83 6.82 6.81 6.80
3.31 H2O
2.47 DMSO-D6
2.46 DMSO-D6
2.46 DMSO-D6
2.45 DMSO-D6
1.18
A (s) 10.52
B (s) 10.27
C (s) 8.14
D (dd) 7.56
E (m) 7.20
F (ddd) 7.13
G (m) 6.99
H (m) 6.83
0.95
0.95
0.95
0.98
2.07
1.05
2.05
2.00
f1 (ppm)

AA263-12

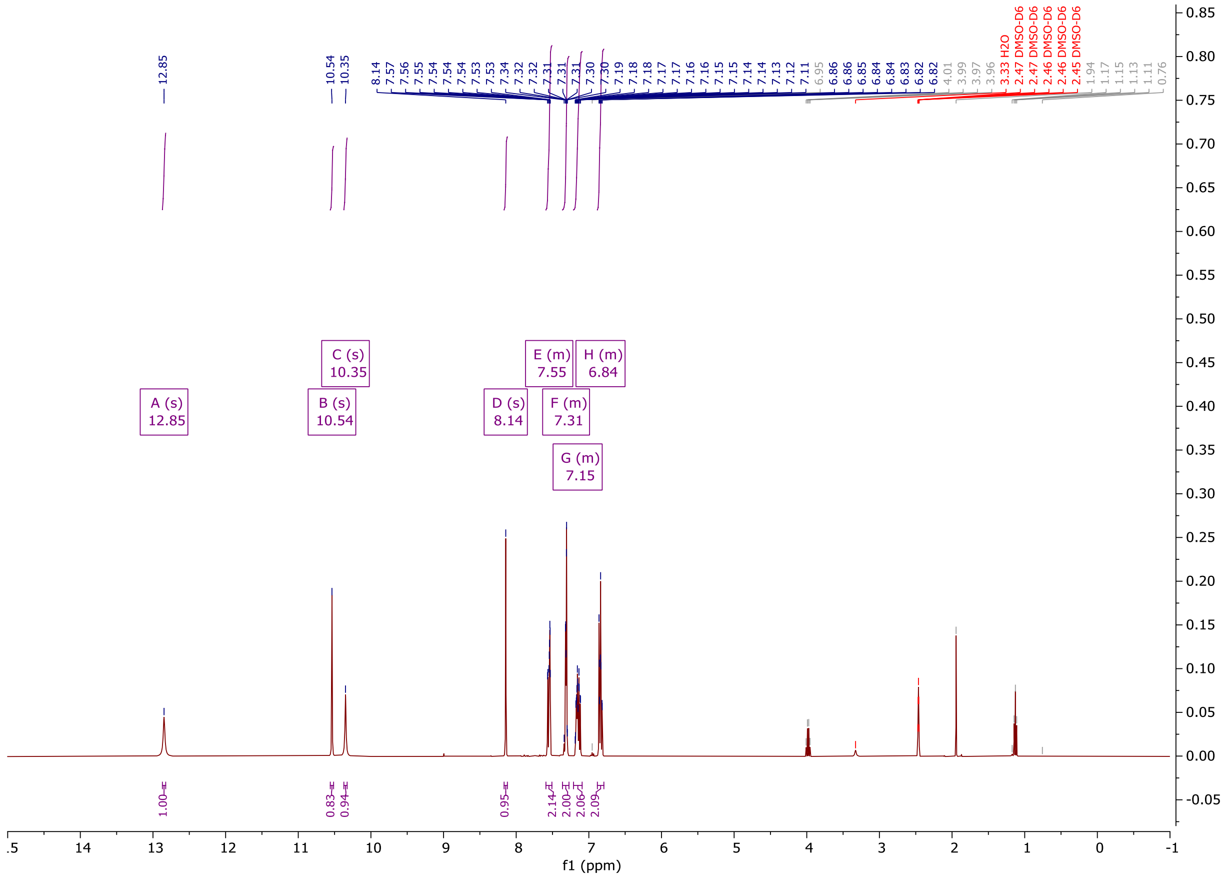

12.85
10.54
10.35
A (s) 12.85
B (s) 10.54
C (s) 10.35
D (s) 8.14
E (m) 7.55
F (m) 7.31
G (m) 7.15
H (m) 6.84
1.00
0.83
0.94
0.95
2.14
2.00
2.06
2.09
3.33 H2O
2.47 DMSO-D6
2.46 DMSO-D6
2.45 DMSO-D6
f1 (ppm)

AA263-13

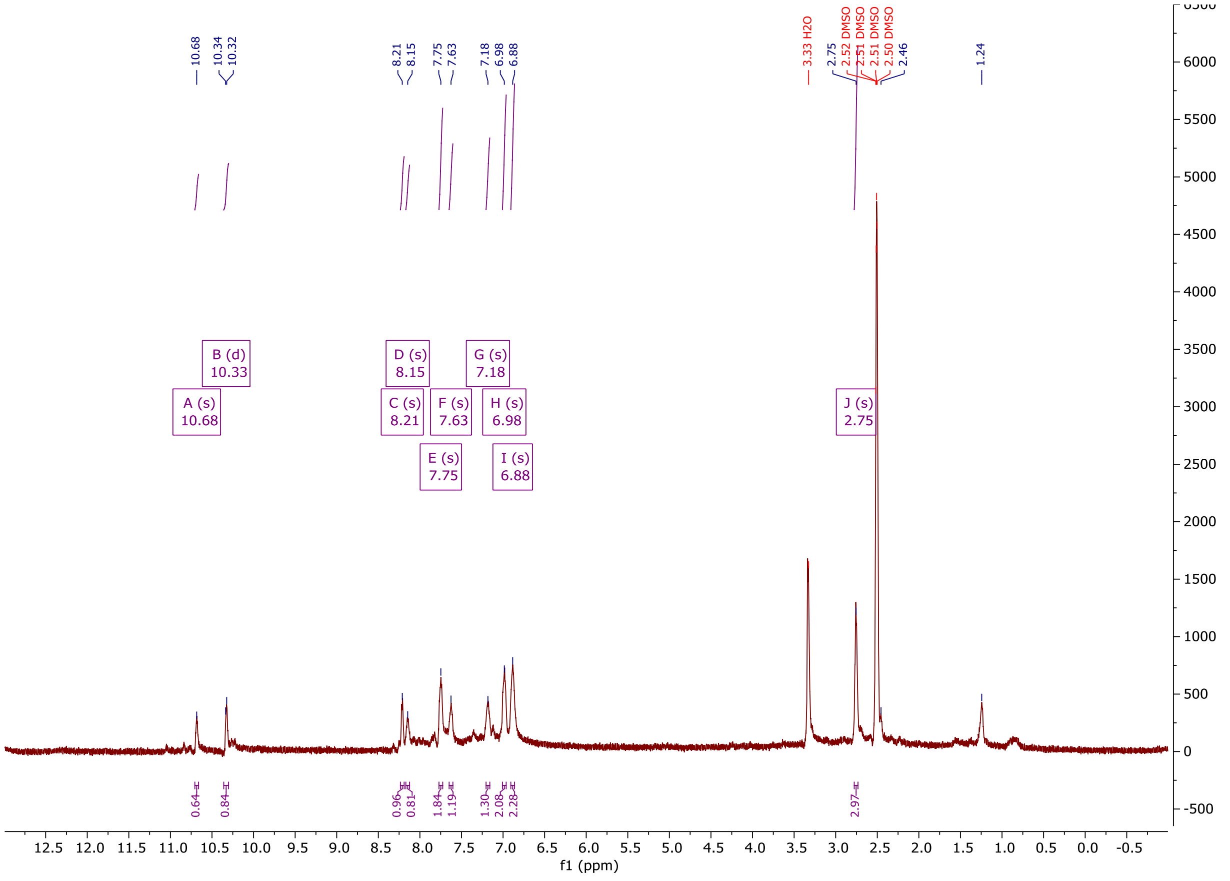

10.68
10.34
10.32
8.21
8.15
7.75
7.63
7.18
6.98
6.88
3.33 H2O
2.75
2.52 DMSO
2.51 DMSO
2.51 DMSO
2.50 DMSO
2.46
1.24
A (s) 10.68
B (d) 10.33
C (s) 8.21
D (s) 8.15
E (s) 7.75
F (s) 7.63
G (s) 7.18
H (s) 6.98
I (s) 6.88
J (s) 2.75
0.64
0.84
0.96
0.81
1.84
1.19
1.30
2.08
2.28
2.97
f1 (ppm)

AA263-14

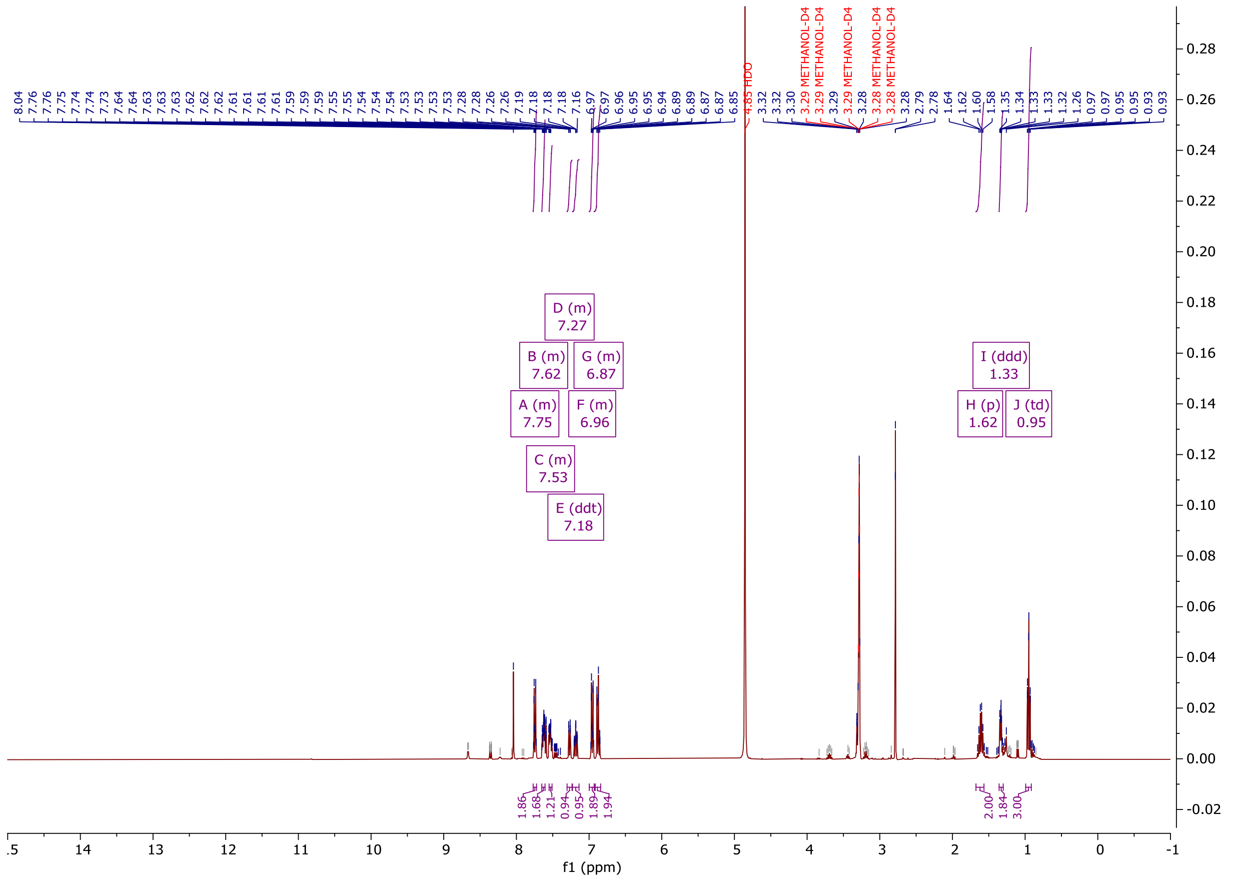
A (m) 7.75
B (m) 7.62
C (m) 7.53
D (m) 7.27
E (ddt) 7.18
F (m) 6.96
G (m) 6.87
H (p) 1.62
I (ddd) 1.33
J (td) 0.95
4.85 HDO
METHANOL-D4
f1 (ppm)

AA263-15

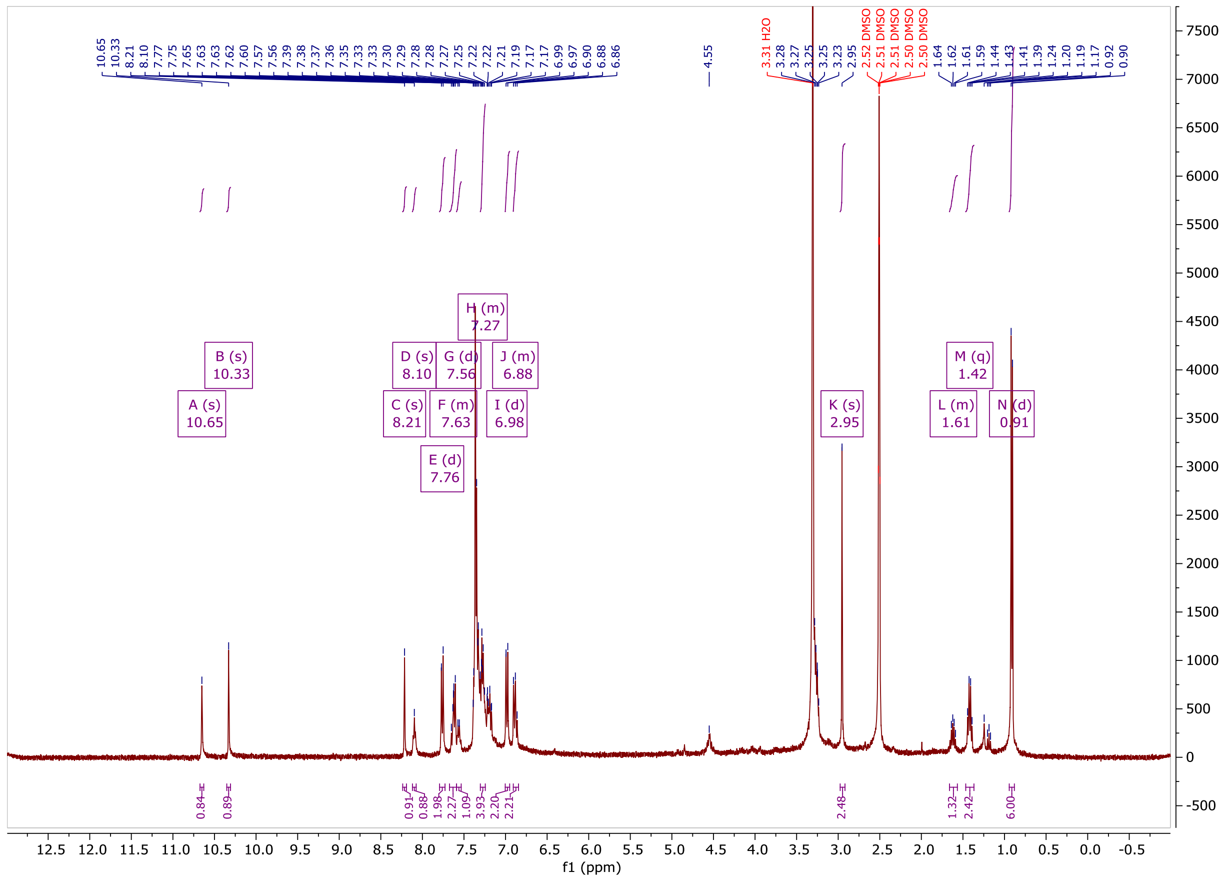
A (s)
10.65
B (s)
10.33
C (s)
8.21
D (s)
8.10
E (d)
7.76
F (m)
7.63
G (d)
7.56
H (m)
7.27
I (d)
6.98
J (m)
6.88
K (s)
2.95
L (m)
1.61
M (q)
1.42
N (d)
0.91
3.31 H2O
2.52 DMSO
2.51 DMSO
2.51 DMSO
2.50 DMSO
2.50 DMSO
f1 (ppm)

AA263-16

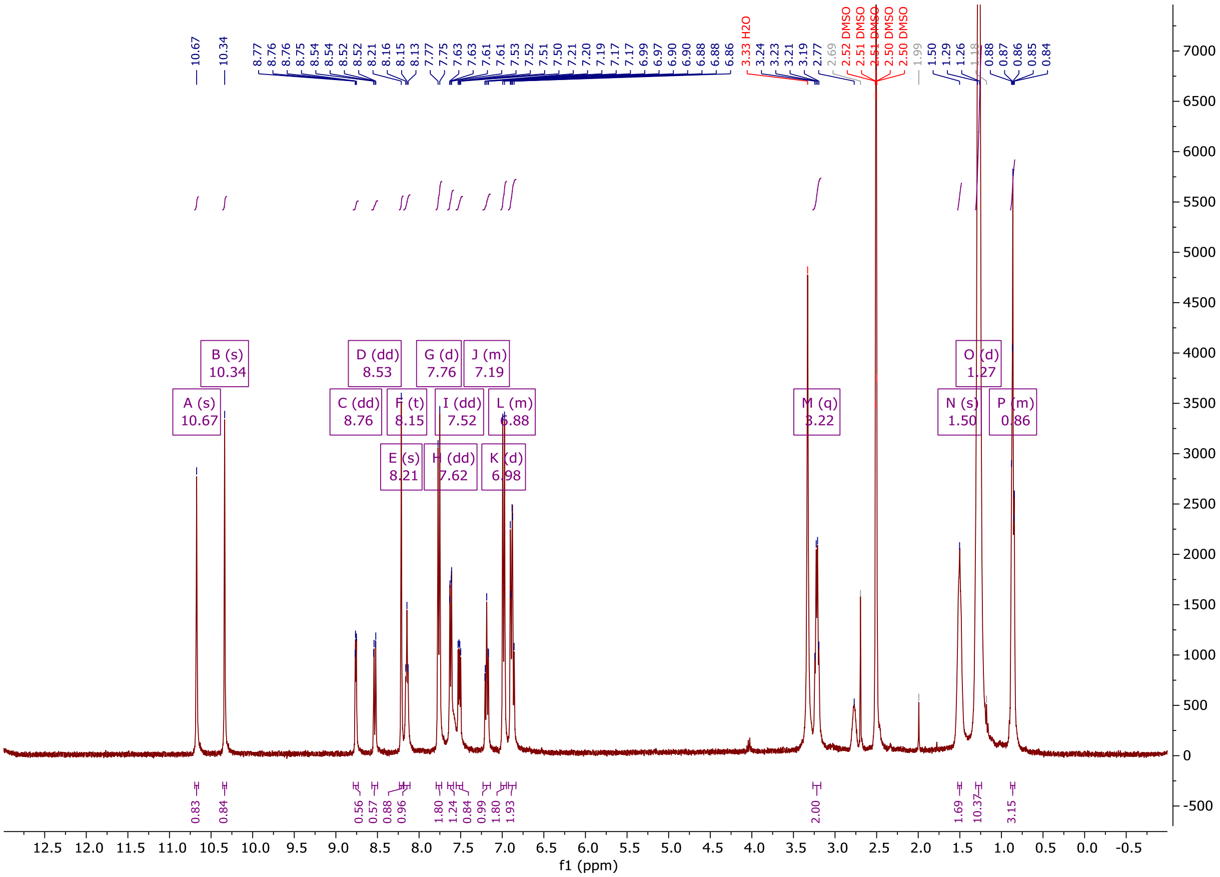

AA263-17

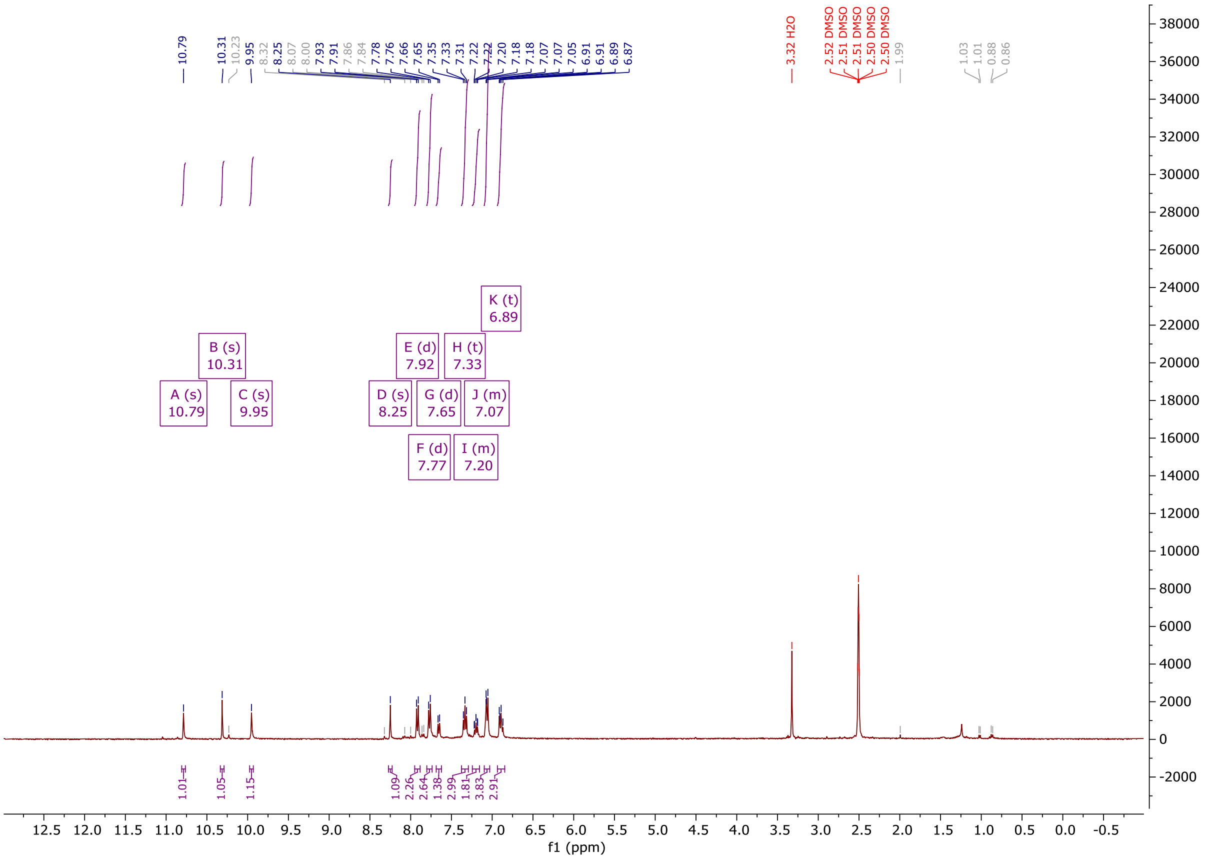

A (s) 10.79
B (s) 10.31
C (s) 9.95
D (s) 8.25
E (d) 7.92
F (d) 7.77
G (d) 7.65
H (t) 7.33
I (m) 7.20
J (m) 7.07
K (t) 6.89
3.32 H2O
2.52 DMSO
2.51 DMSO
2.51 DMSO
2.50 DMSO
2.50 DMSO
f1 (ppm)

AA263-18

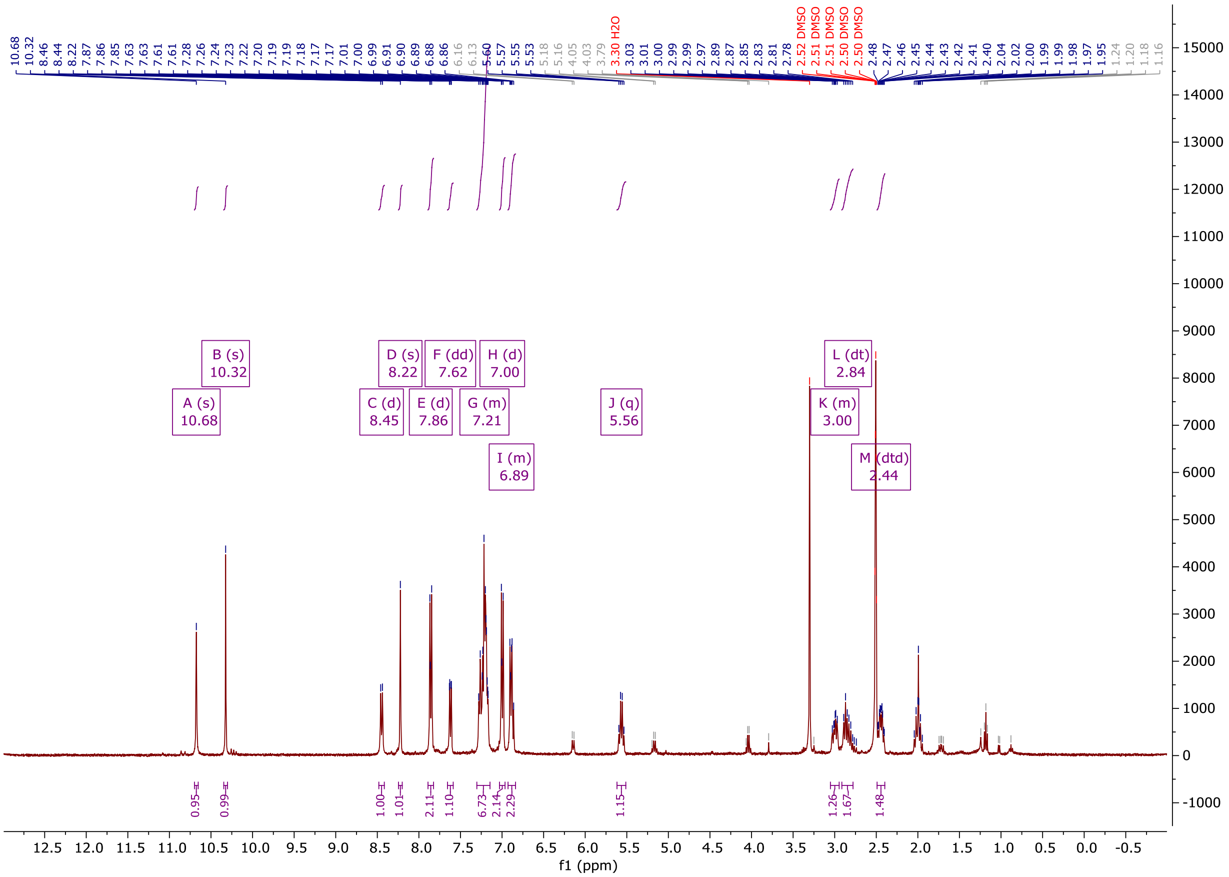

AA263-19

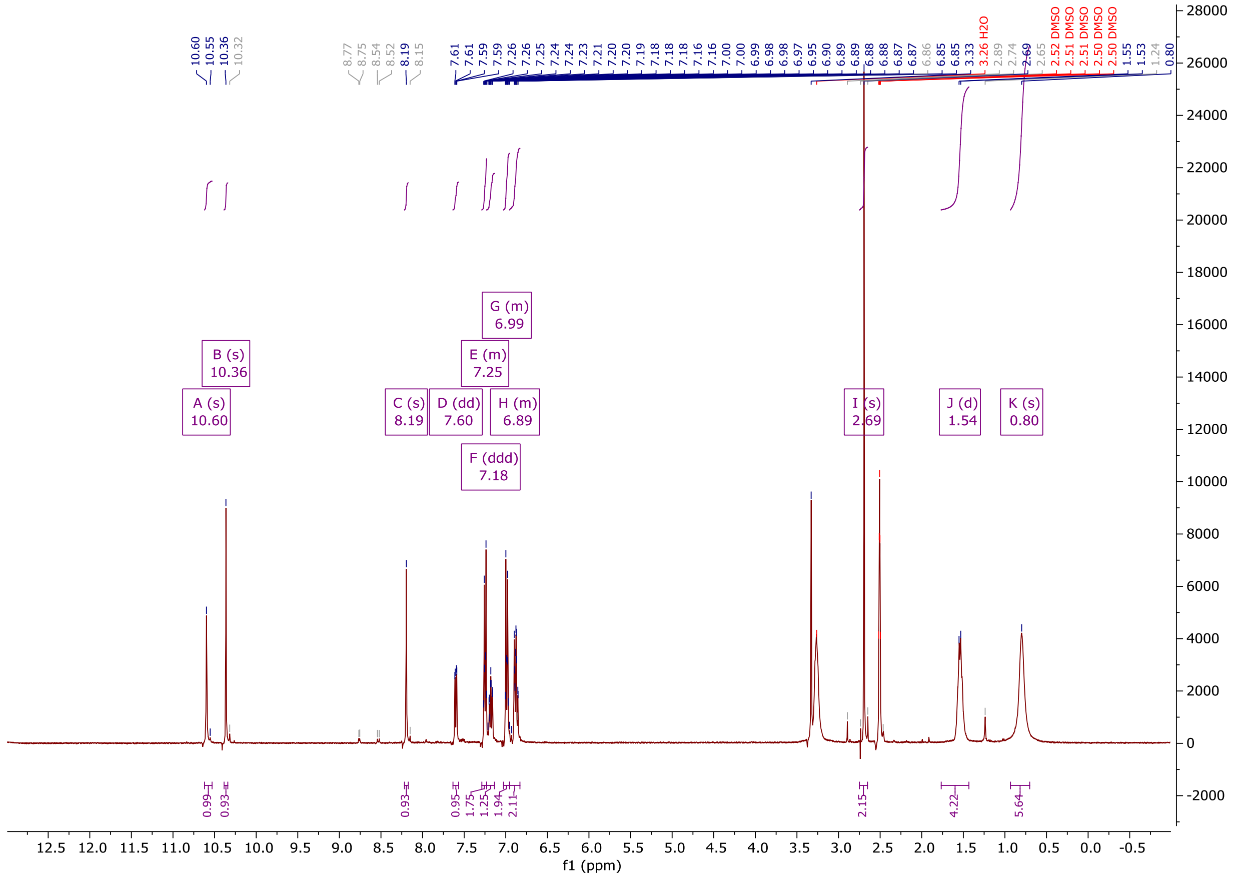

A (s) 10.60
B (s) 10.36
C (s) 8.19
D (dd) 7.60
E (m) 7.25
F (ddd) 7.18
G (m) 6.99
H (m) 6.89
I (s) 2.69
J (d) 1.54
K (s) 0.80
3.26 H2O
2.52 DMSO
2.51 DMSO
2.51 DMSO
2.50 DMSO
2.50 DMSO
f1 (ppm)

AA263-20

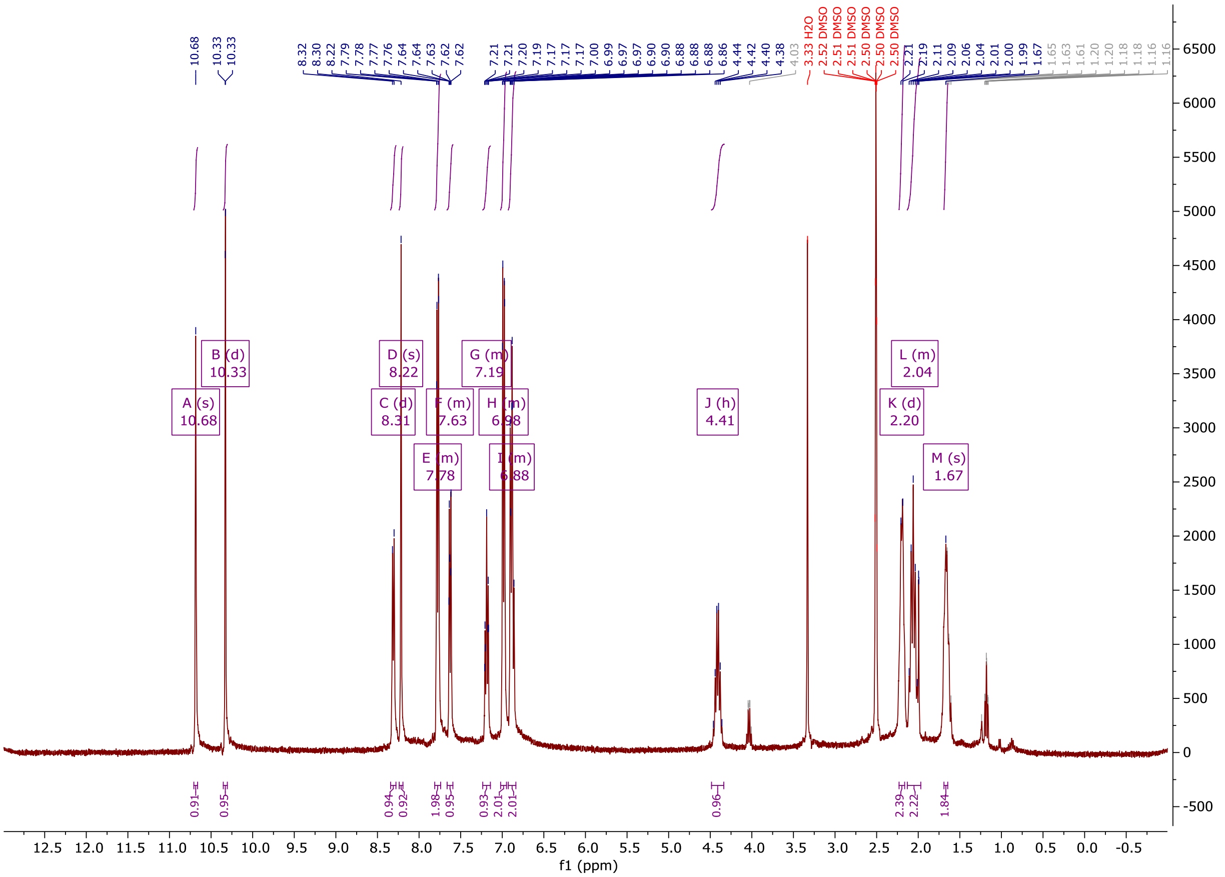

10.68
10.33
10.33
8.32
8.30
8.22
7.79
7.78
7.77
7.76
7.64
7.64
7.63
7.62
7.62
7.21
7.21
7.20
7.19
7.17
7.17
7.17
7.00
6.99
6.97
6.97
6.90
6.90
6.88
6.88
6.86
4.44
4.42
4.40
4.38
4.03
3.33 H2O
2.52 DMSO
2.51 DMSO
2.51 DMSO
2.50 DMSO
2.50 DMSO
2.50 DMSO
2.21
2.19
2.11
2.09
2.06
2.04
2.01
2.00
1.99
1.67
1.65
1.63
1.61
1.20
1.18
1.18
1.16
1.16
A (s)
10.68
B (d)
10.33
C (d)
8.31
D (s)
8.22
E (m)
7.78
F (m)
7.63
G (m)
7.19
H (m)
6.98
I (m)
6.88
J (h)
4.41
K (d)
2.20
L (m)
2.04
M (s)
1.67
0.91
0.95
0.94
0.92
1.98
0.95
0.93
2.01
2.01
0.96
2.39
2.22
1.84
f1 (ppm)
